# Supplementary material for: R-loop editing by DNA cytosine deaminase APOBEC3B modulates the activity of oestrogen receptor enhancers
Source: Nat Commun. 2026 Feb 18;17:2887. doi: 10.1038/s41467-026-69679-4 (PMC13031881; doi:10.1038/s41467-026-69679-4)
Supplement: Supplementary file 1 — Supplementary Information [file 41467_2026_69679_MOESM1_ESM.pdf]

Supplemental Materials

Contents:

1. Supplemental Figures 1-21, and legends
2. Supplemental Table 1
3. Supplemental Reference

Supplemental Figures

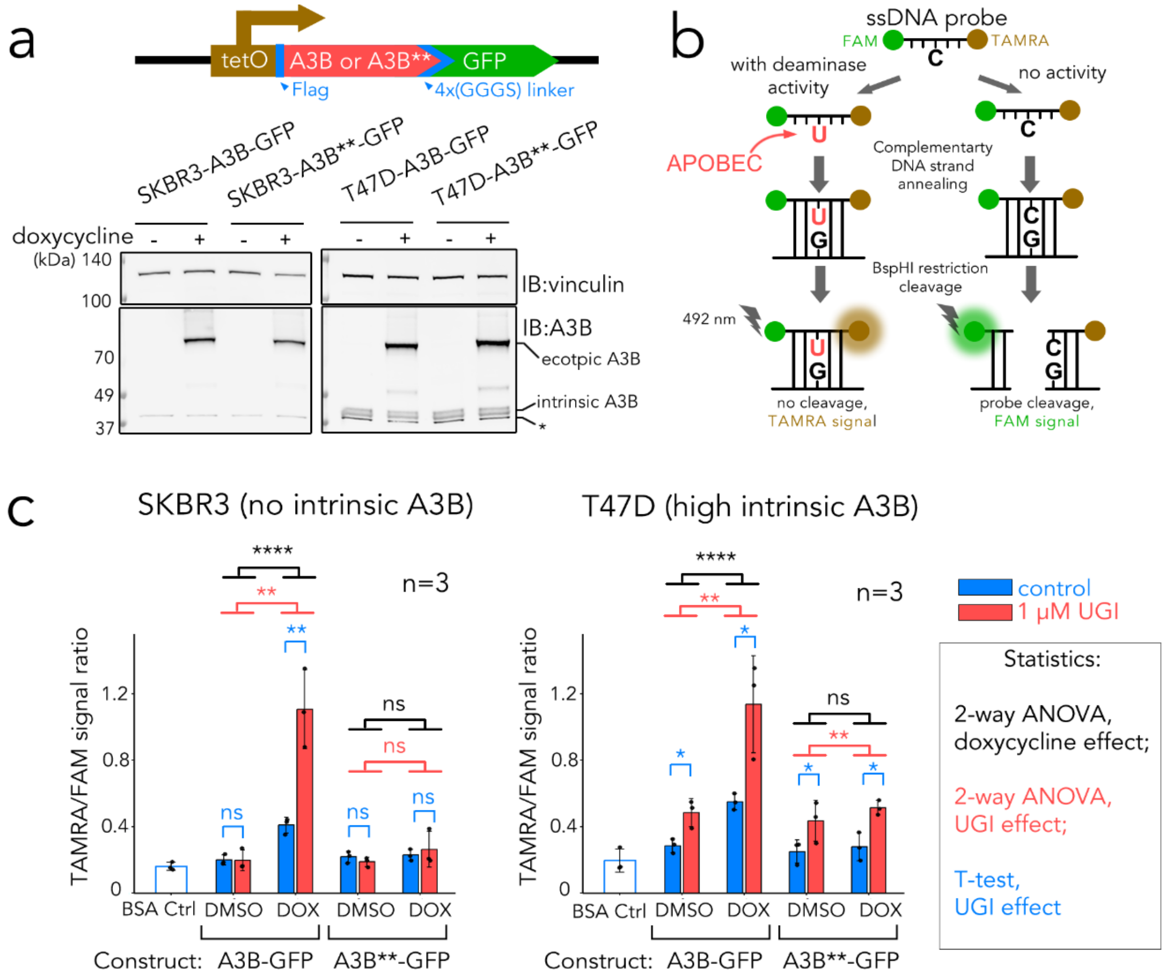

Supplementary Figure 1: Characterisation of lentiviral inducible A3B constructs used in this study.

a) Schematic representation of the lentiviral inducible system for expression of the A3B–GFP fusion protein (top) and immunoblot analysis of A3B-null human SKBR3 breast cancer cells and high A3B-expressing human T47D breast cancer cells with or without induction using 1 µg/mL doxycycline for 24 h. A3B** indicates the A3B variant containing E68Q/E255Q mutations. Asterisk (*) marks a non-specific immunoblotting band. The immunoblot shown is representative of two independent experiments.

b) Schematic of the cellular DNA deaminase activity assay using the BspHI sensor[1] (adapted from Zhang et al., 2022 [1]).

c) DNA deaminase activity in lysates from SKBR3 and T47D cells induced with or without A3B–GFP (1 µg/mL doxycycline, 24 h), as measured by the method depicted in (B). Endogenous uracil-DNA glycosylase (UDG) activity was inhibited in parallel reactions by

addition of UGI peptide (final concentration 1 μM). Statistical analysis: two-sided two-way ANOVA was used to evaluate the effects of doxycycline and UGI (and their interaction), and two-sided Student's t-tests were used for pairwise comparisons evaluating the effect of UGI peptide where indicated. Significance levels: **** $p < 0.0001$; ** $p < 0.01$; * $p < 0.05$; ns $p > 0.05$. For SKBR3 cells, two-way ANOVA in A3B–GFP–expressing cells showed a significant effect of doxycycline ($p = 1.3 \times 10^{-3}$) and of UGI ($p = 0.5 \times 10^{-5}$). The effect of UGI peptide in doxycycline-induced A3B–GFP cells was further evaluated by Student's t-test ($p = 0.7 \times 10^{-3}$). For T47D cells, two-way ANOVA in A3B–GFP–expressing cells showed a significant effect of doxycycline ($p = 0.2 \times 10^{-3}$) and of UGI ($p = 0.9 \times 10^{-4}$). In A3B**–GFP–expressing cells, two-way ANOVA showed a significant effect of UGI peptide ($p = 0.3 \times 10^{-3}$). Student's t-tests evaluating the effect of UGI in A3B–GFP cells showed $p = 0.2 \times 10^{-3}$ for DMSO-treated and $p = 0.2 \times 10^{-3}$ for doxycycline-treated samples. Student's t-tests evaluating the effect of UGI in A3B**–GFP cells showed $p = 0.2 \times 10^{-3}$ for DMSO-treated and $p = 0.1 \times 10^{-3}$ for doxycycline-treated samples.

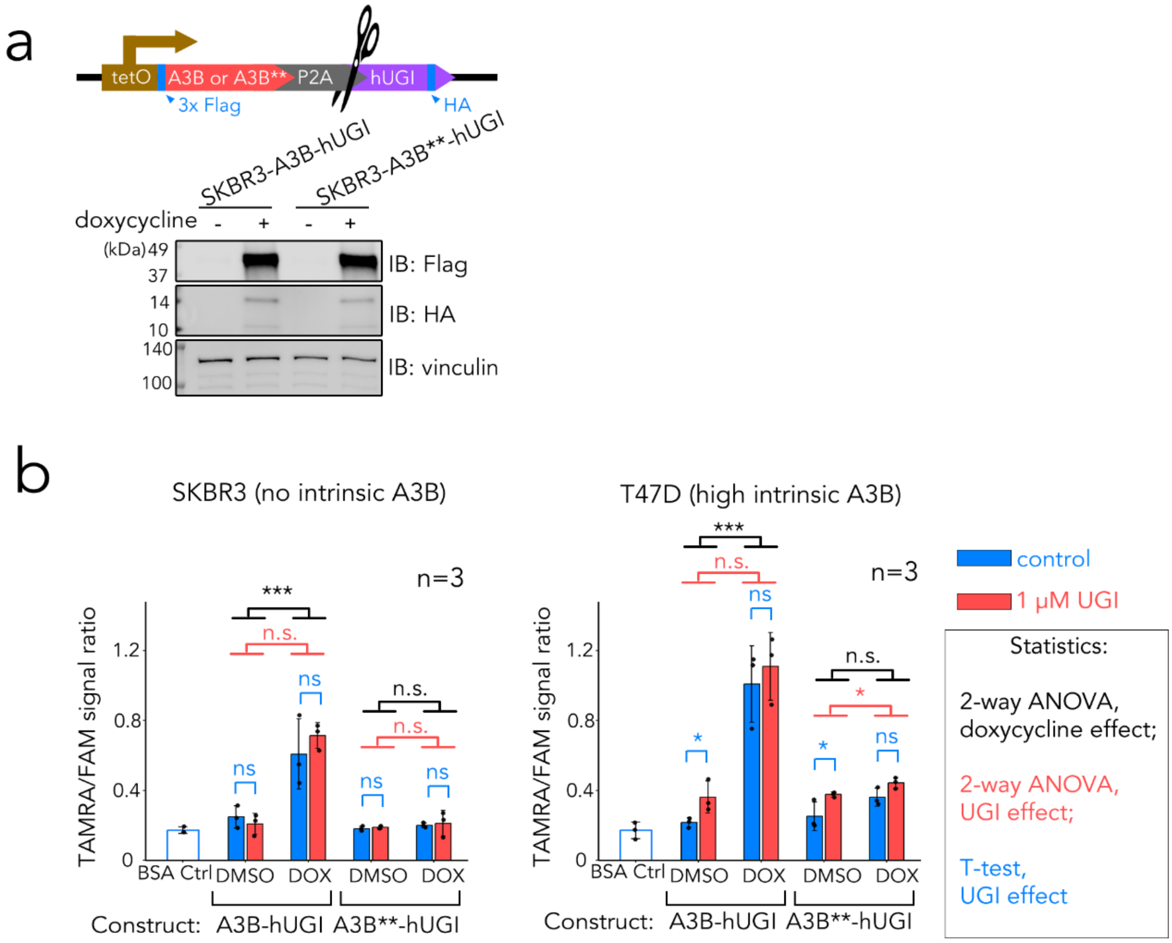

Supplementary Figure 2: Functional suppression of UDG/UNG activity by lentiviral hUGI in cells.

a) Immunoblot analysis of SKBR3 cells transduced with inducible A3B(A3B**)-hUGI constructs, confirming expression of the indicated proteins. Cells were treated with 1 µg/mL doxycycline for 24 h. The immunoblot shown is representative of two independent experiments.

b) DNA deaminase activity in lysates from SKBR3 and T47D cells induced with or without A3B(A3B**)-hUGI (1 µg/mL doxycycline, 24 h). Excess UGI peptide (final concentration 1 µM) was added to lysates, and assays were performed in parallel. Values represent the mean of $n = 3$ independent experiments; error bars indicate standard deviation (SD). Statistical analysis (two-sided): for SKBR3 cells, two-way ANOVA in A3B–hUGI–expressing cells: doxycycline effect, $p = 1.9 \times 10^{-4}$. For T47D cells, two-way ANOVA in A3B–hUGI–expressing cells: doxycycline effect, $p = 2.0 \times 10^{-4}$; in A3B**–hUGI–expressing cells: UGI peptide effect, $p = 0.017$. Two-sided Student's t-tests evaluating the effect of UGI peptide: DMSO-treated A3B–hUGI cells, $p = 0.04$; DMSO-treated A3B**–

hUGI cells, $p = 0.04$. Significance levels: **** $p < 0.0001$; ** $p < 0.01$; * $p < 0.05$; ns $p > 0.05$.

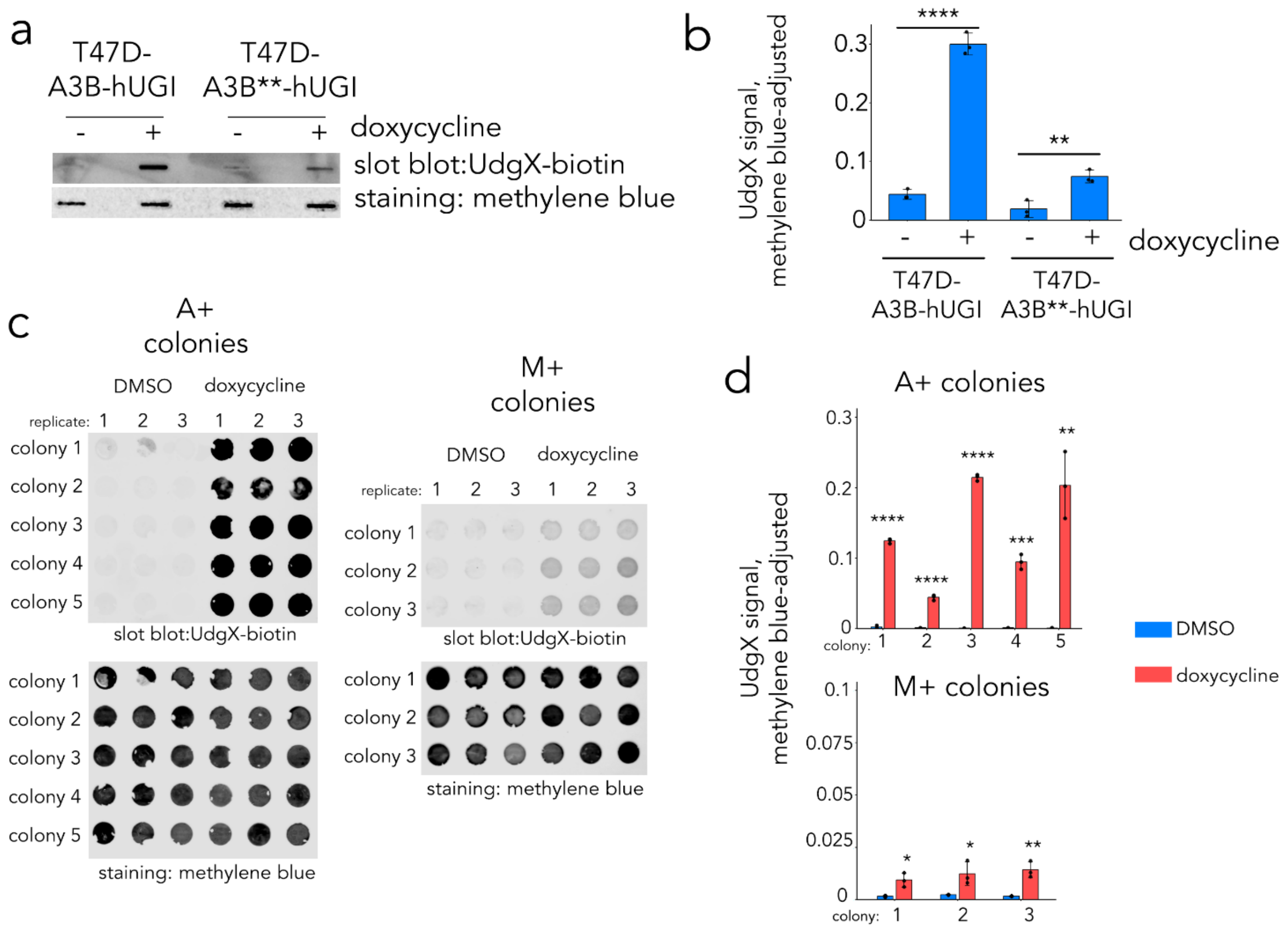

Supplementary Figure S3: Characterisation of DNA-incorporated uridine in A3B–hUGI inducible T47D cell lines.

a) Representative slot blots of genomic DNA from the indicated cell lines. DNA-incorporated uridine was detected using biotinylated UdgX protein followed by streptavidin–HRP; total DNA was visualised by methylene blue staining. The slot blot shown is representative of three independent experiments.

b) Quantification of uracil incorporation in the indicated cells, based on UdgX slot blot signal intensity normalised to total DNA (methylene blue). Two-sided Student's t-test evaluating the effect of doxycycline: $p = 2.74 \times 10^{-5}$ in A3B–hUGI cells and $p = 5.8 \times 10^{-3}$ in A3B**–hUGI cells.

c) Dot blots of genomic DNA isolated from individual colonies of the indicated cell lines, probed as in (a). The dot blots shown are representative of three independent experiments.

d) Quantification of uridine signal in (c), normalised to total DNA. Two-sided Student's t-test evaluating the effect of doxycycline: $p = 7 \times 10^{-7}$, 3.8×10^{-5}, 1.3×10^{-7}, 1×10^{-4}, and 1.7×10^{-3} for A^+ colonies 1–5; p = 0.016, 0.036, and 5.1×10^{-3} for M^+ colonies 1–3.

For panels (b) and (d), values represent the mean of $n = 3$ independent experiments; error bars indicate SD. *, **, ***, and **** indicate $p < 0.05$, < 0.01, $< 10^{-3}$, and $< 10^{-4}$, respectively. UdgX, uracil-DNA glycosylase from *Mycobacterium smegmatis* (biotinylated); HRP, horseradish peroxidase.

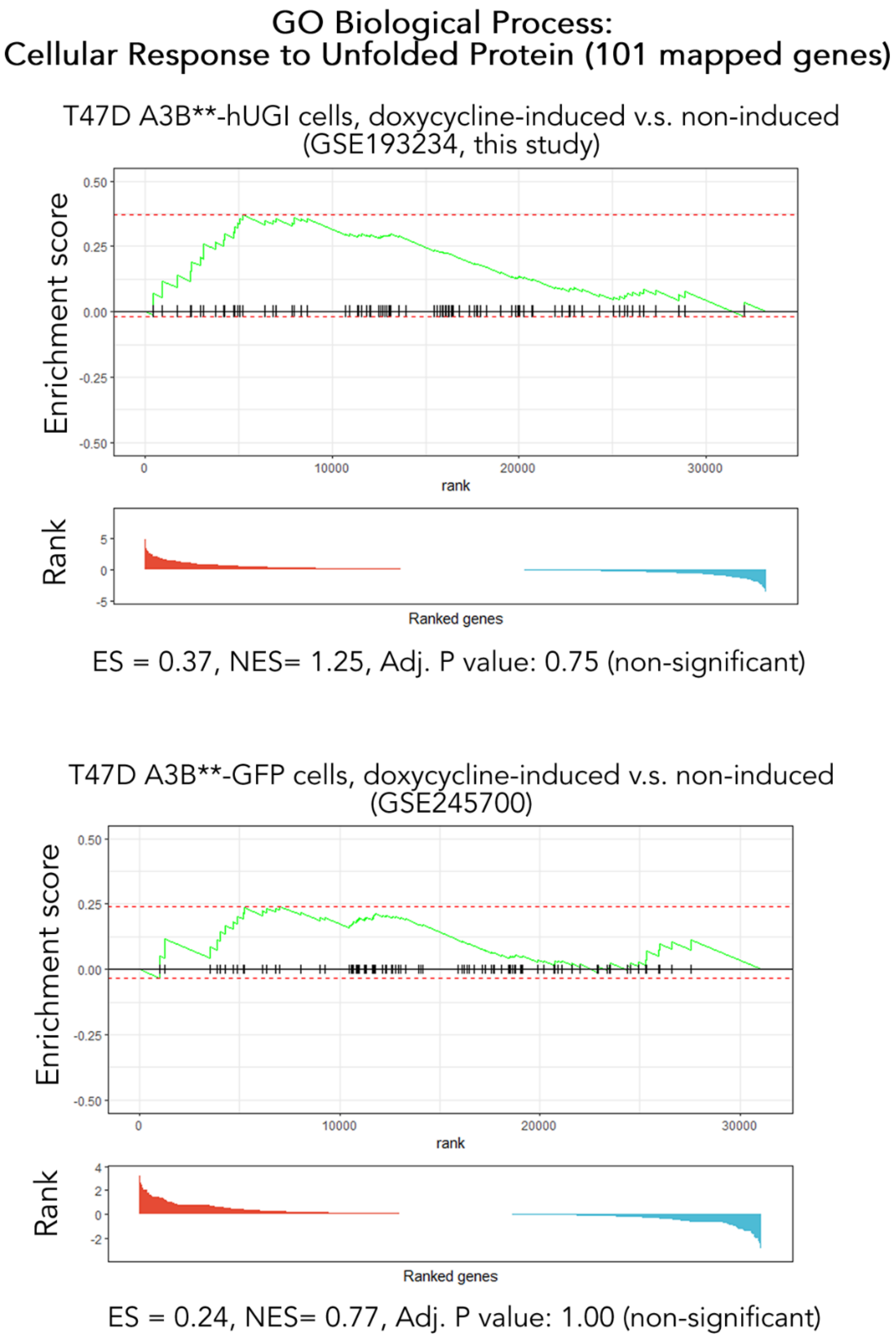

Supplementary Figure 4: Gene set enrichment analysis (GSEA) evaluating the potential unfolded protein response following induction of A3B**(E68Q/E255Q) in T47D cells.

Leading-edge plots for 101 genes associated with the unfolded protein response, curated under the Gene Ontology (GO) Biological Process (BP) category. The dataset includes RNA-seq results from T47D cells induced with or without the A3B**–hUGI construct (generated in this study; GSE193234) or the A3B**–GFP construct (from a previous study; GSE245700). Enrichment scores (ES) and normalised enrichment scores (NES) are indicated at the bottom of each leading-edge plot.

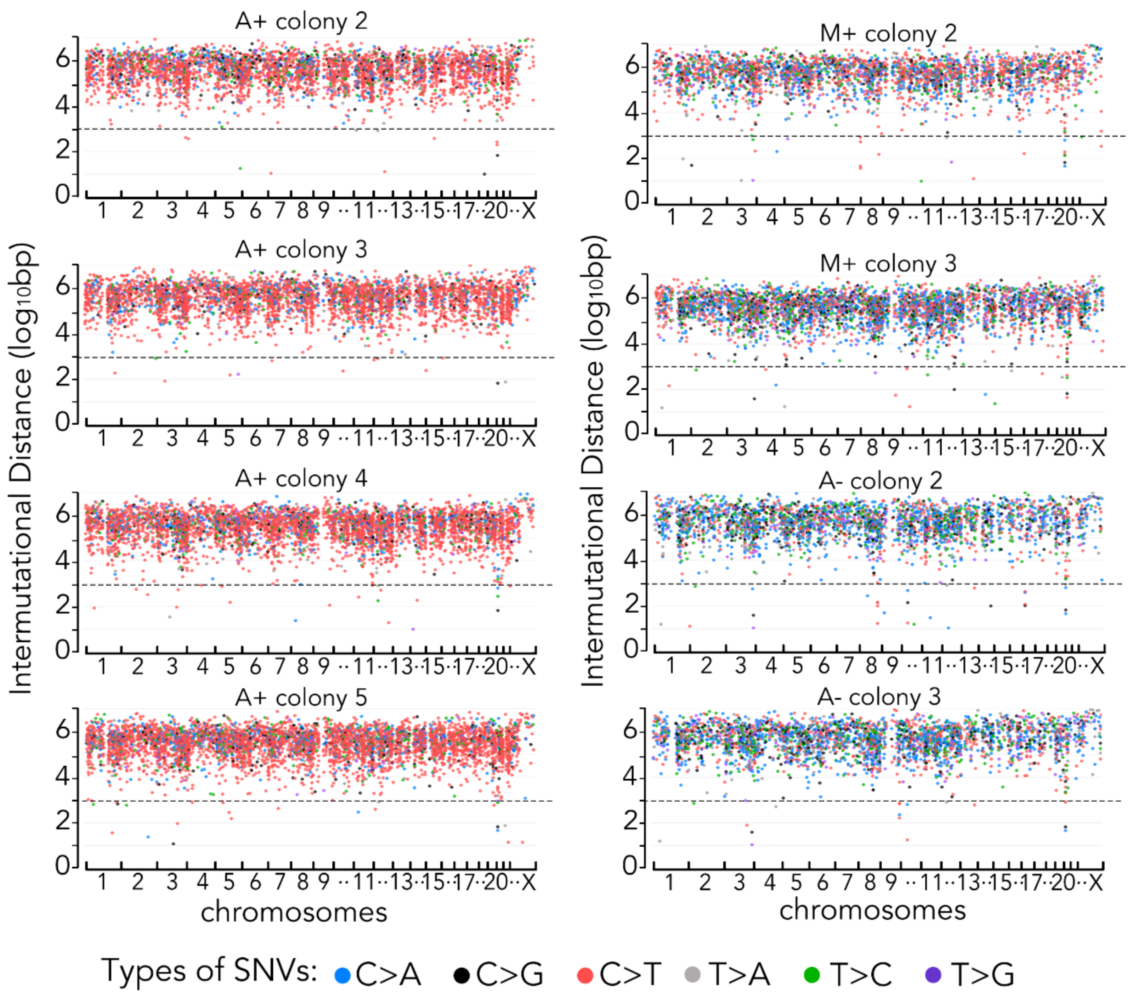

Supplementary Figure 5: Capture of A3B DNA editing sites in BER-deficient cell models. Waterfall plot of intermutational distance (IMD) of each mutation identified in the indicated colonies. Dotted line denotes IMD ≤ 10^3 bp.

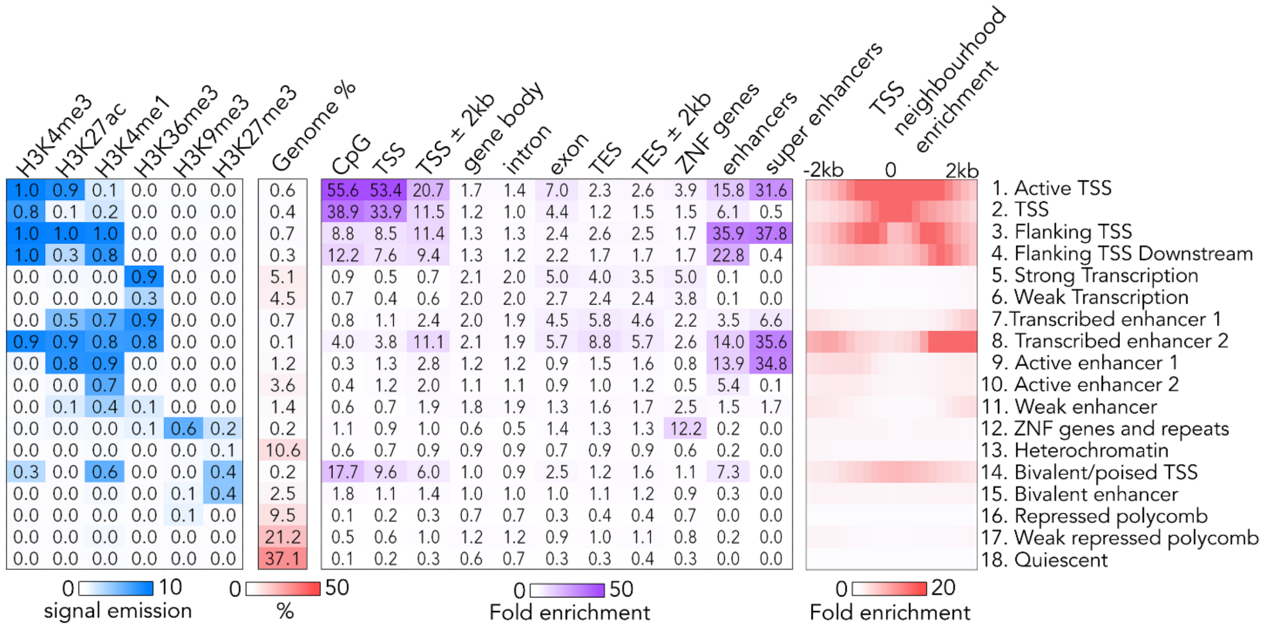

Supplementary Figure 6: Analysis of chromatin states for SNVs using ChromHMM model. 18-state ChromHMM model used in this study, which was derived from six epigenetic marks. For validation, enrichment scores for ChromHMM-curated known genomic regions as well as enrichment scores for TSS neighbourhood regions are shown. In addition, enhancer segments from Enhancer Atlas [2] and super enhancer segments from Chan et al., 2018 [3] were included for the enrichment analysis.

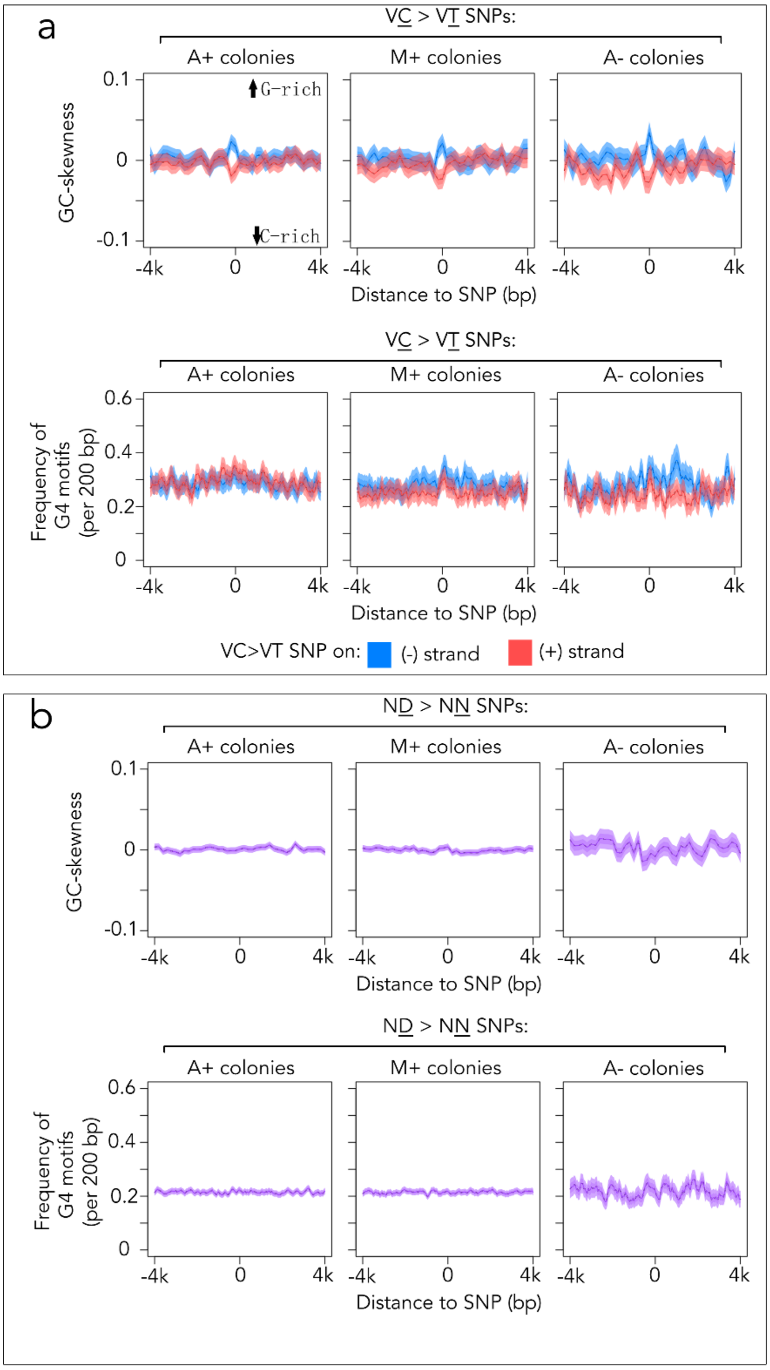

Supplementary Figure 7: Sequence traits flanking SNVs identified in WGS analysis.

a) Profiles of GC skewness and frequency of G-quadruplex (G4) motifs in regions flanking the VC>VT SNP identified in indicated colonies.

b) Profiles of GC skewness and frequency of G-quadruplex (G4) motifs in regions flanking the ND>NN SNP identified in indicated colonies.

Line denotes average value, and shaded area indicates 95% CI.

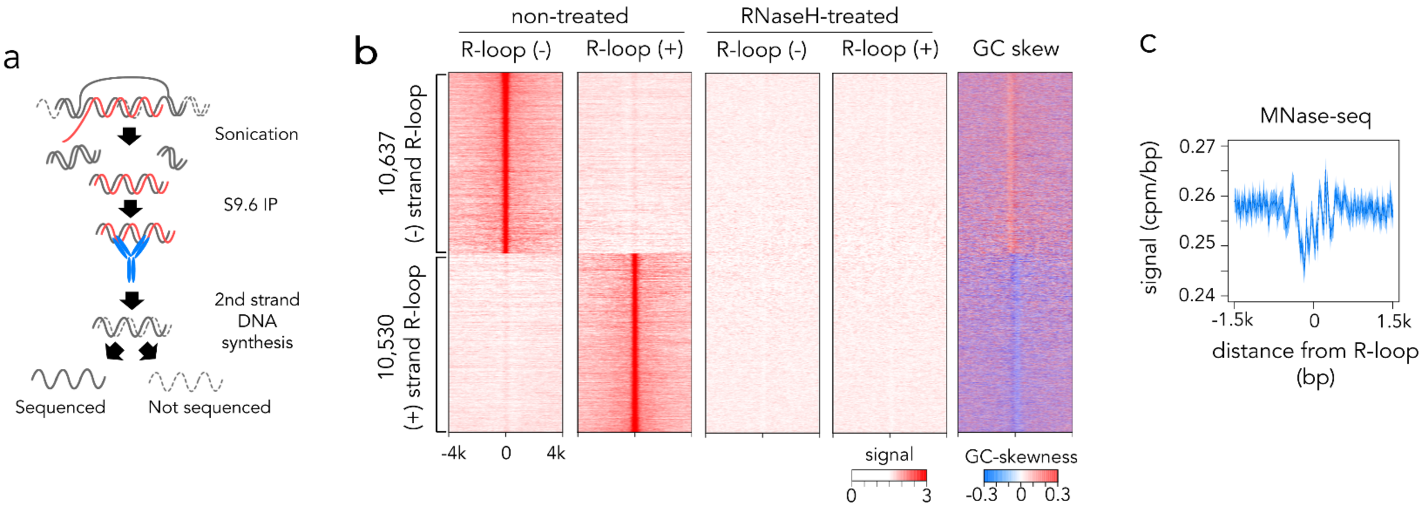

Supplementary Figure 8: Mapping R-loops in T47D genome using strand-specific DRIP-seq.

a) Schematic of the ssDRIP-seq experiment carried out in this study.

b) Heat maps of signal from ssDRIP-seq and GC skew score in regions flanking high-confidence R-loops identified by MACS2 programme.

c) Profile of MNase-seq signals in region flanking centre of R-loops. Line denotes average value and shaded areas 95% CI.

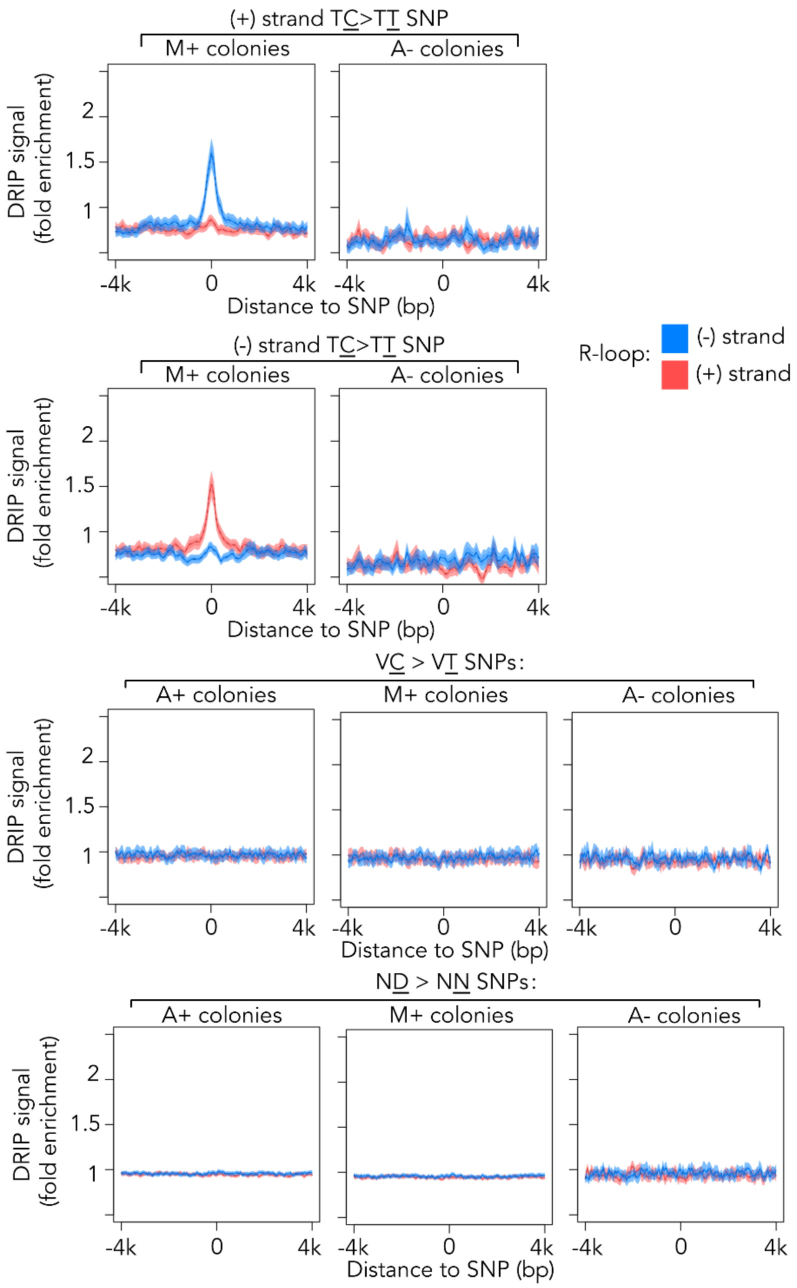

Supplementary Figure 9: Signals of ssDRIP-seq flanking SNVs identified from WGS analysis. Signal profiles showing ssDRIP-seq signals in regions flanking the TC>TT SNP or VC>VT SNP identified in indicated colonies on indicated strand. Line denotes average value and shaded areas 95% CI.

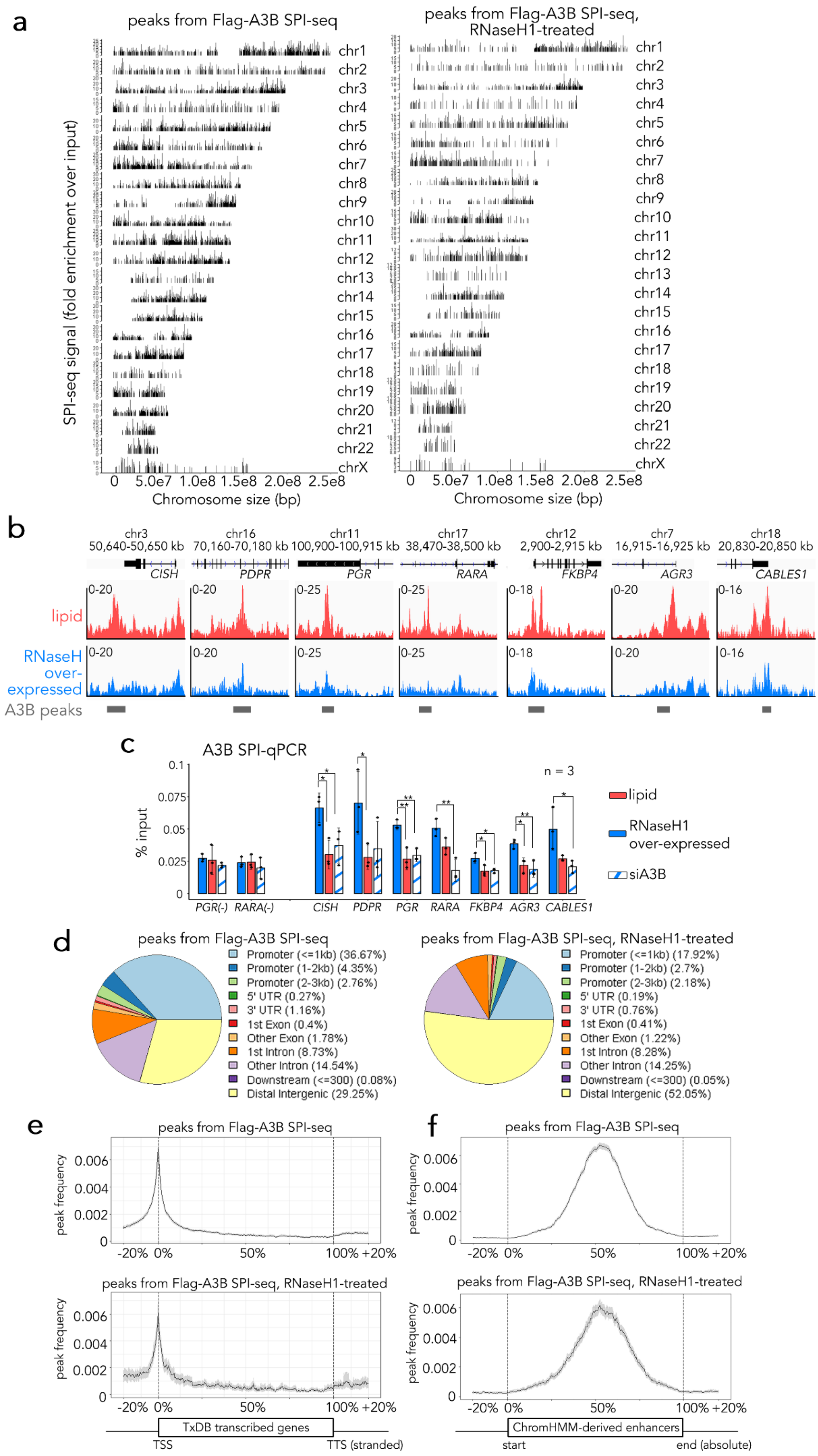
a
peaks from Flag-A3B SPI-seq
peaks from Flag-A3B SPI-seq, RNaseH1-treated
SPI-seq signal (fold enrichment over input)
chr1
chr2
chr3
chr4
chr5
chr6
chr7
chr8
chr9
chr10
chr11
chr12
chr13
chr14
chr15
chr16
chr17
chr18
chr19
chr20
chr21
chr22
chrX
0 5.0e7 1.0e8 1.5e8 2.0e8 2.5e8
Chromosome size (bp)
b
chr3 50,640-50,650 kb
chr16 70,160-70,180 kb
chr11 100,900-100,915 kb
chr17 38,470-38,500 kb
chr12 2,900-2,915 kb
chr7 16,915-16,925 kb
chr18 20,830-20,850 kb
CISH
PDPR
PGR
RARA
FKBP4
AGR3
CABLES1
lipid
RNaseH over-expressed
A3B peaks
0-20
0-25
0-18
0-16
c
A3B SPI-qPCR
% input
n = 3
lipid
RNaseH1 over-expressed
siA3B
PGR(-) RARA(-) CISH PDPR PGR RARA FKBP4 AGR3 CABLES1
d
peaks from Flag-A3B SPI-seq
Promoter (<=1kb) (36.67%)
Promoter (1-2kb) (4.35%)
Promoter (2-3kb) (2.76%)
5' UTR (0.27%)
3' UTR (1.16%)
1st Exon (0.4%)
Other Exon (1.78%)
1st Intron (8.73%)
Other Intron (14.54%)
Downstream (<=300) (0.08%)
Distal Intergenic (29.25%)
peaks from Flag-A3B SPI-seq, RNaseH1-treated
Promoter (<=1kb) (17.92%)
Promoter (1-2kb) (2.7%)
Promoter (2-3kb) (2.18%)
5' UTR (0.19%)
3' UTR (0.76%)
1st Exon (0.41%)
Other Exon (1.22%)
1st Intron (8.28%)
Other Intron (14.25%)
Downstream (<=300) (0.05%)
Distal Intergenic (52.05%)
e
f
peak frequency
-20% 0% 50% 100% +20%
TxDB transcribed genes
TSS
TTS (stranded)
ChromHMM-derived enhancers
start
end (absolute)

Supplementary Figure 10: Mapping and validation of A3B genomic occupancy using SPI-seq.

a) Coverage plot for peaks from SPI-seq experiments over T47D genome.

b) Representative signal tracks for Flag-A3B SPI-seq at identified A3B binding sites. Data represents fold change of signal (read count per million reads, CPM) over input control.

c) Validation of signal specificity for SPI-seq experiments using an antibody against endogenous A3B. Data represent mean values from n = 3 biological repeats, and error bars indicate SD. Two-tailed Student's t-test: for the effect of RNaseH1 overexpression, $p = 0.017$ (CISH), 0.0491 (PDPR), 7.1×10^{-3} (PGR), 0.038 (FKBP4), 0.012 (AGR3), and 0.073 (CABLES1); for the effect of A3B depletion, $p = 0.048$ (CISH), 2.5×10^{-3} (PGR), 0.016 (FKBP4), 6.3×10^{-3} (RARA), 8.4×10^{-3} (AGR3), and 0.041 (CABLES1). *: $p \leq 0.05$; **: $p \leq 0.01$.

d) Genomic annotation of peaks from SPI-seq experiments. UCSC hg19 genome database was used for generating annotations.

e) Frequency of SPI-seq peaks over transcribed gene body. Line denotes average value and shaded area for 95% CI.

f) Frequency of SPI-seq peaks over T47D's enhancers derived from the ChromHMM model in this study. Line denotes average value and shaded area for 95% CI.

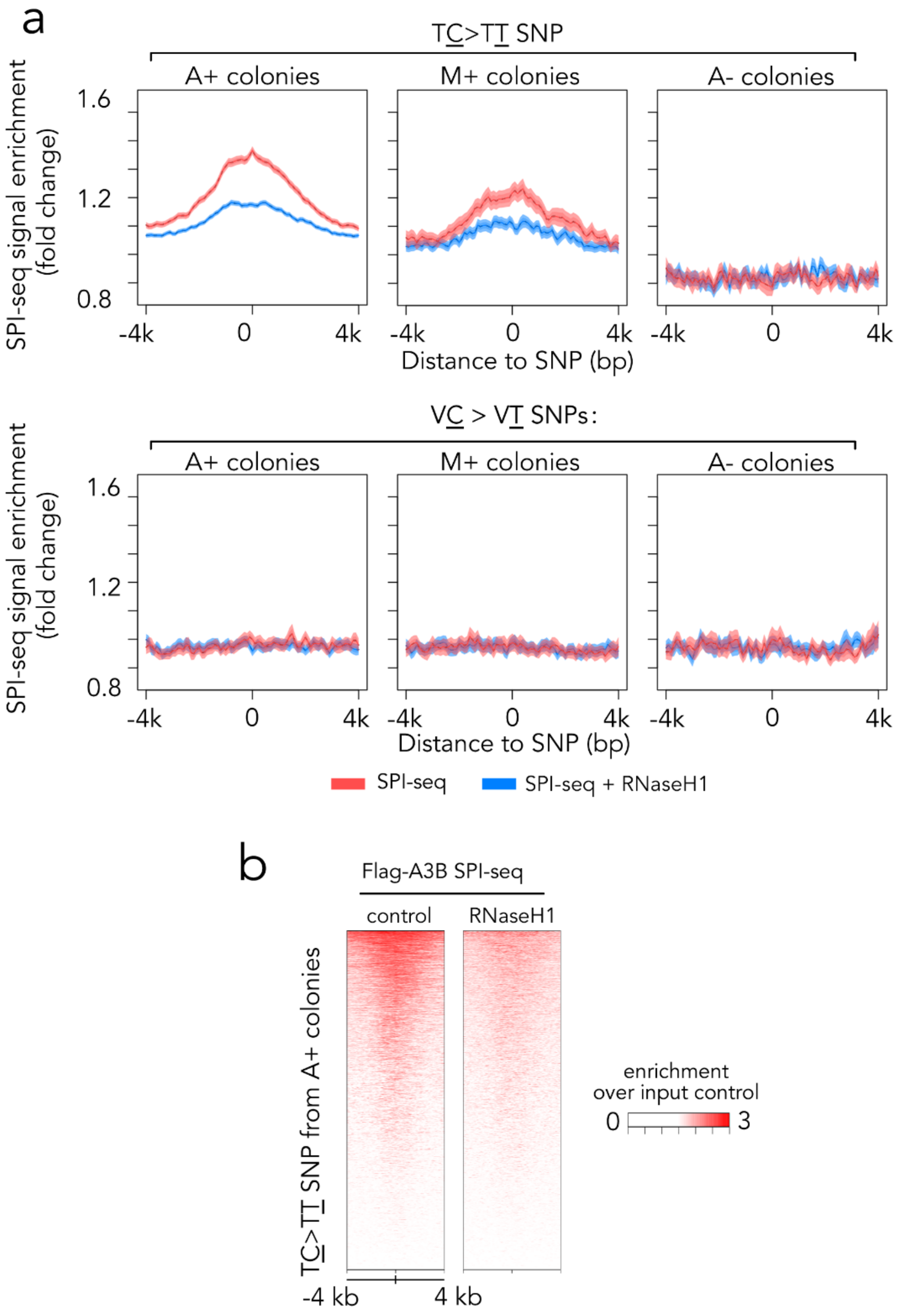

Supplementary Figure 11: Levels of A3B genomic occupancy at WGS-derived SNPs.

a) Signal profiles for Flag-A3B ChIP-seq or SPI-seq signal in regions flanking TC>TT SNP or VC>VT SNP identified in indicated colonies. Line denotes average value and shaded areas 95% CI.

b) Heat maps showing SPI-seq signal in regions flanking TC>TT SNP identified in A+ colonies.

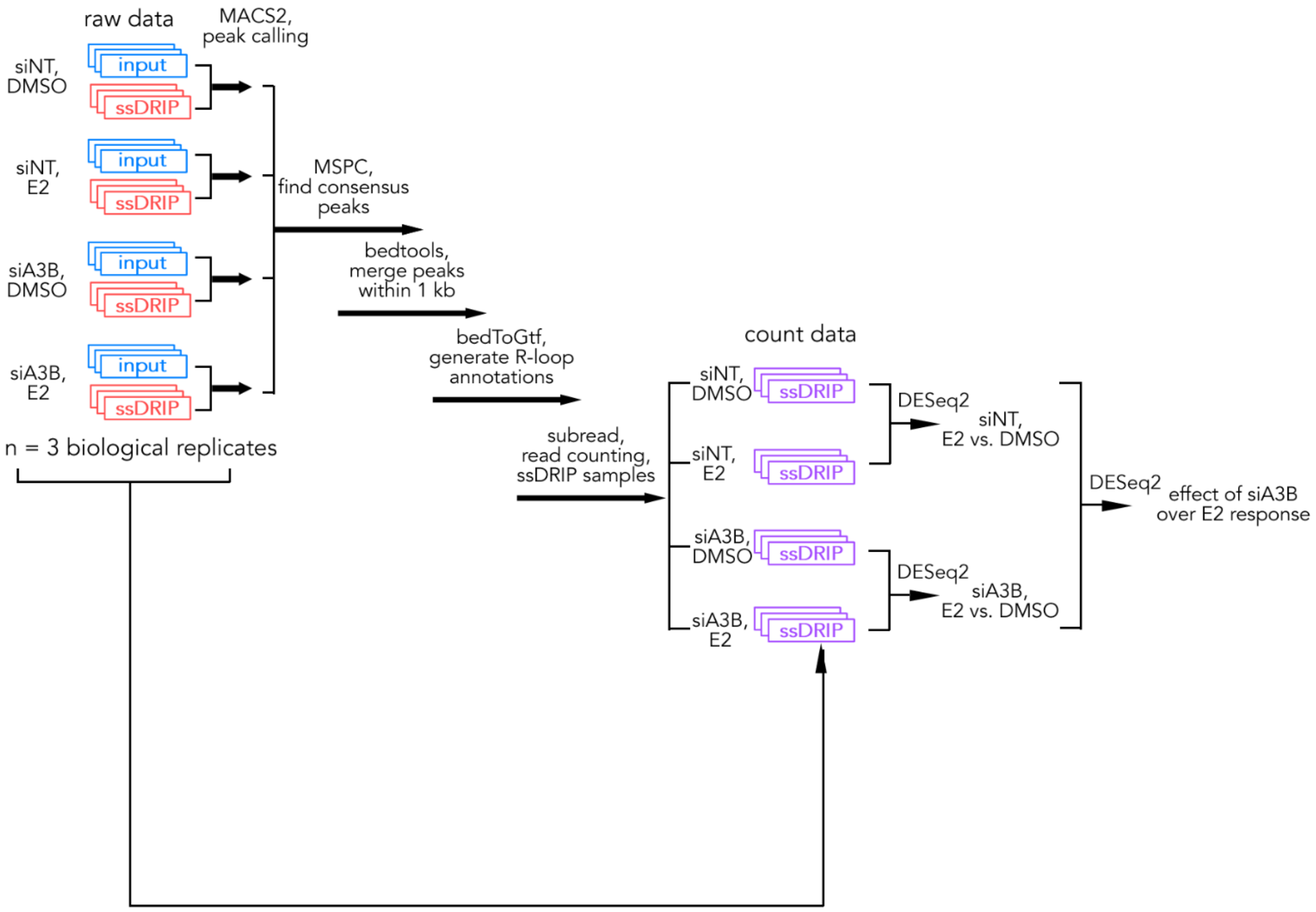

Supplementary Figure 12: Diagram depicting the data analysis procedures of quantitative ssDRIP-seq.

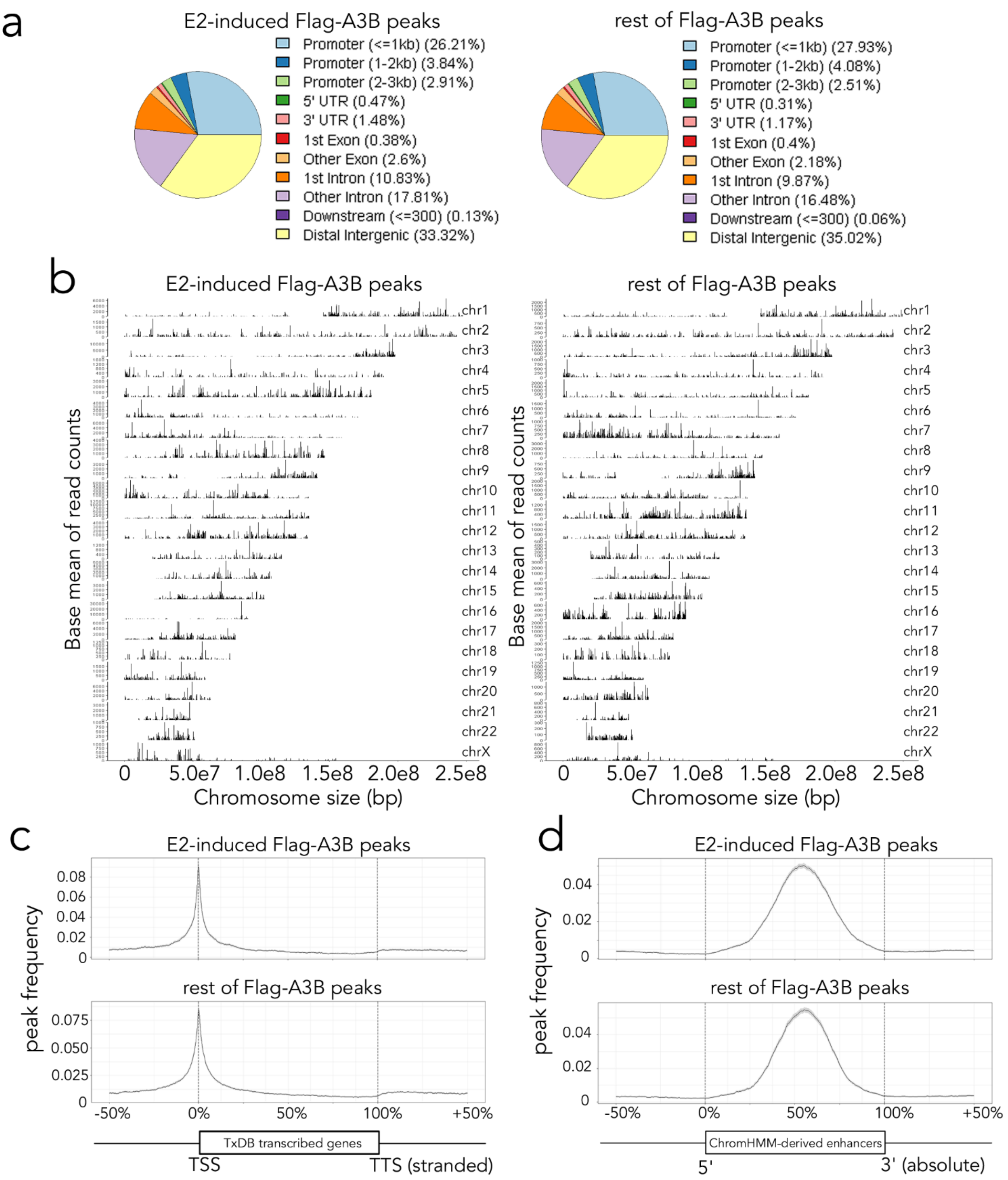

Supplementary Figure 13: Genome-wide analysis of the quantitative results from Flag-A3B SPI-seq.

a) Genomic annotation of peaks from quantitative SPI-seq experiments. UCSC hg19 genome database was used for generating annotations. For identification of E2-induced SPI-seq peaks, criteria of fold change ≥ 1.5 and FDR ≤ 0.05 was used.

b) Coverage plot for peaks from quantitative SPI-seq experiments over T47D genome.

c) Frequency of SPI-seq peaks over transcribed gene body. Line denotes average value and shaded area for 95% CI.

d) Frequency of SPI-seq peaks over ChromHMM-derived enhancers. Line denotes average value and shaded area for 95% CI.

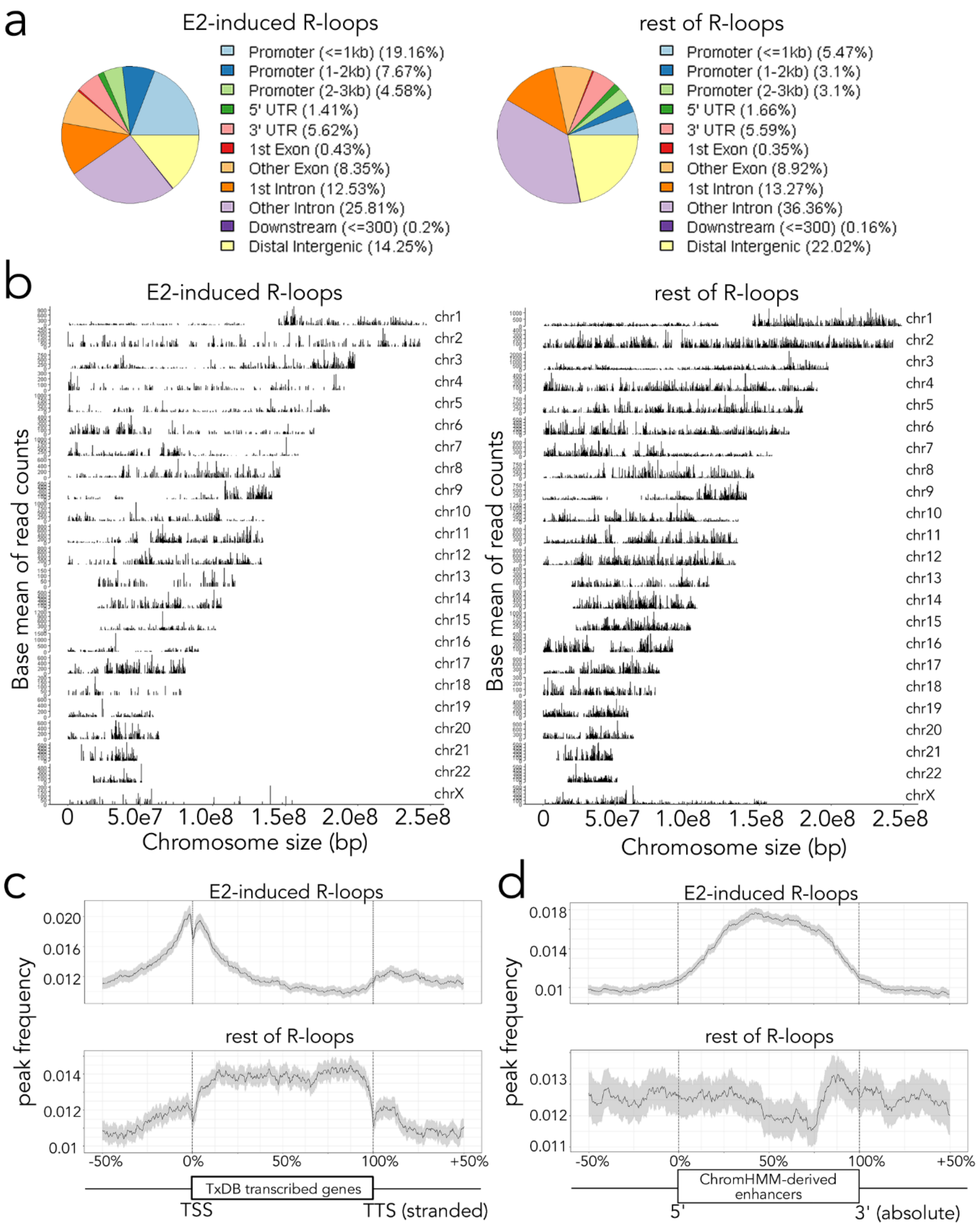

Supplementary Figure 14: Genome-wide analysis of the quantitative results from ssDRIP-seq.

a) Genomic annotation of peaks from quantitative ssDRIP-seq experiments. UCSC hg19 genome database was used for generating annotations. For identification of E2-induced ssDRIP-seq peaks, criteria of fold change ≥ 1.5 and FDR ≤ 0.05 was used.

b) Coverage plot for peaks from quantitative ssDRIP-seq experiments over T47D genome.

c) Frequency of ssDRIP-seq peaks over transcribed gene body. Line denotes average value and shaded area for 95% CI.

d) Frequency of ssDRIP-seq peaks over ChromHMM-derived enhancers. Line denotes average value and shaded area for 95% CI.

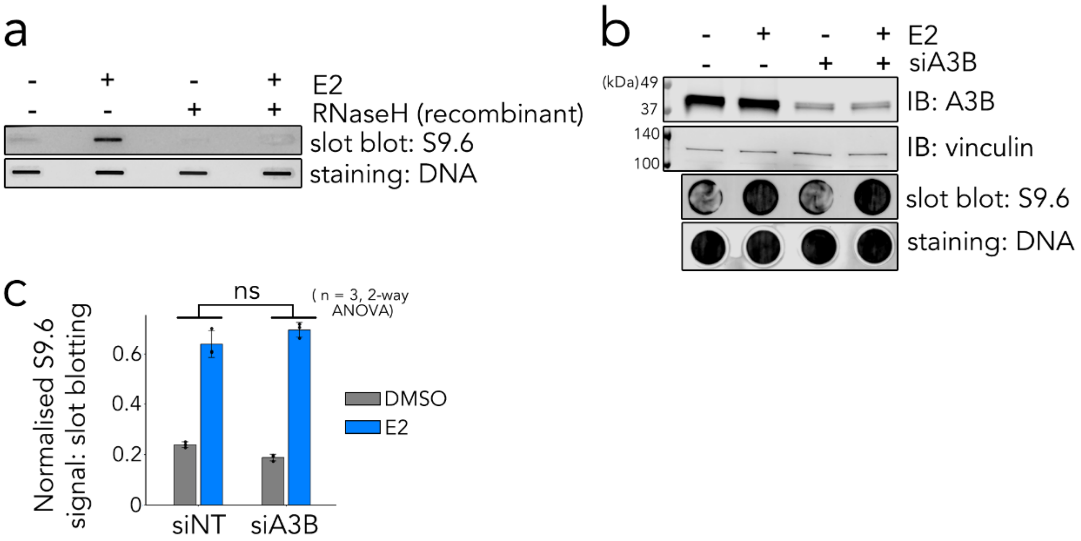

Supplementary Figure 15: A3B depletion had minimal impact on R-loop formation upon E2 induction in T47D cells.

a) Representative slot blots of gDNA samples from T47D cells with or without two-hour stimulation by 100 nM E2. To confirm specificity of S9.6 antibody, gDNA samples treated overnight with recombinant RNaseH were included as control. The slot blot shown is representative of two independent experiments.

b) Representative dot blots and immunoblots of samples from T47D cells treated with or without two-hour stimulation by 100 nM E2, and with or without depletion of A3B by siRNA. The blots shown are representative of three independent experiments.

c) Bar graph depicting data analysis of (B). Data represent average value of methylene blue-adjusted S9.6 signal from three biological repeats and error bars indicate SD. ns denotes non-significant from two-way ANOVA assessing size effect of A3B depletion on E2 response.

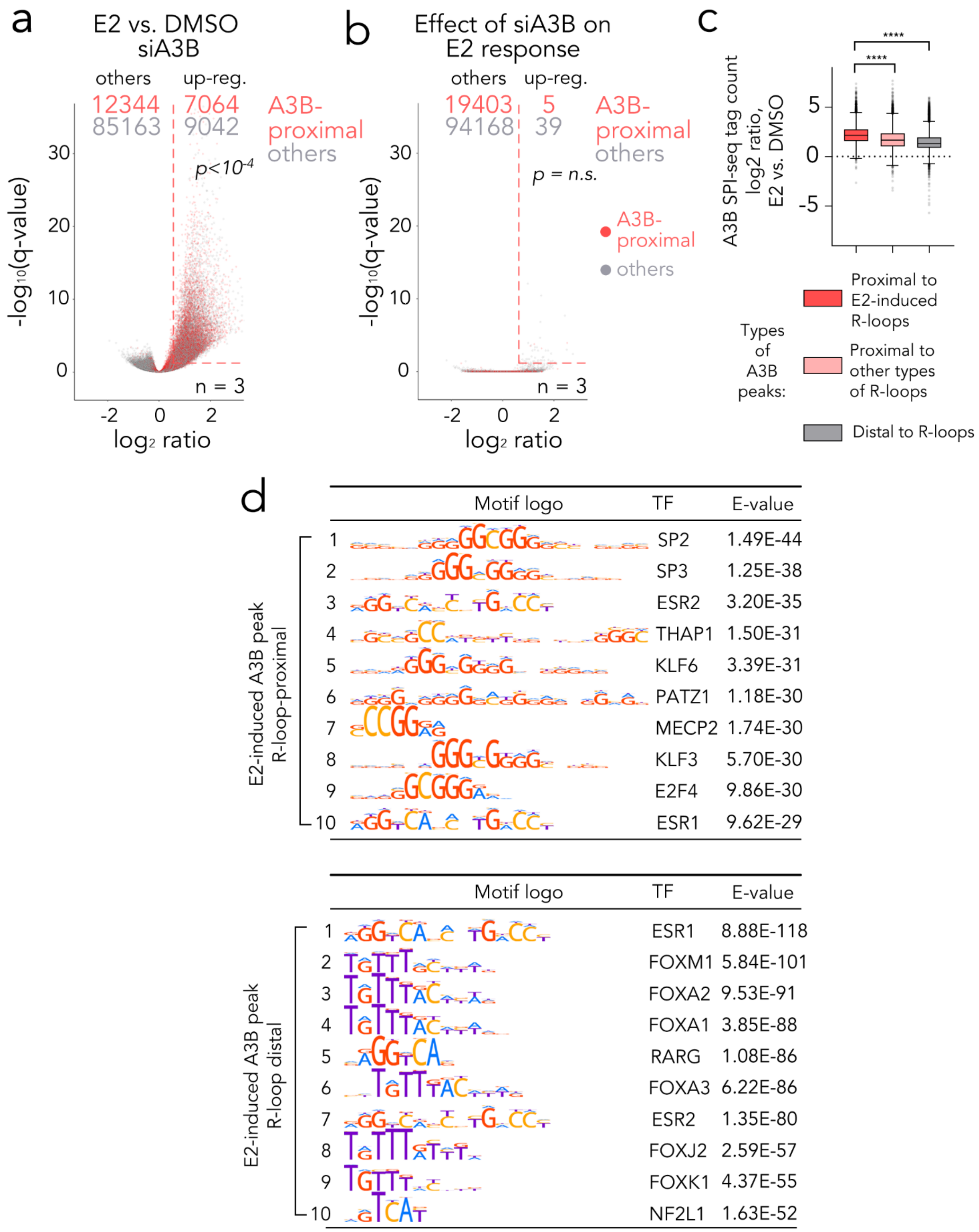

Supplementary Figure 16: E2-induced R-loop formation was not dependent on A3B but might alter A3B binding pattern.

a) Volcano plot summarising the effect of two-hour stimulation by E2 on R-loop formation in A3B siRNA-treated cells, determined by ssDRIP-seq using DESeq2 statistics. p value represents statistical significance by χ^2 test of non-random association between R-loop or A3B proximity and response to estradiol.

b) Volcano plot summarising the effect of A3B depletion on E2 response of R-loop level, determined by ssDRIP-seq using DESeq2 statistics. For D and E, A3B-proximal R-loops are coloured in red. Criteria for up-regulated R-loops are defined as FDR ≤ 0.05 with fold change ≥ 1.5 and is labelled with red dotted line. p value represent statistical significance by χ^2 test of non-random association between R-loop or A3B proximity and response to estradiol.

c) Tukey boxplots showing log2 ratio of SPI-seq signals for Flag-A3B binding sites in response to estradiol. Data derived from two biological replicates for each condition using edgeR. ****: $p \leq 10^{-4}$, one-way ANOVA evaluating effect size between groups.

d) Top ranking transcription factor motifs enriched in R-loop-proximal (left) or R-loop-distal (right) Flag-A3B sites that were induced by E2. Enrichment scores (E-values) were derived by AME from MEME suite using shuffled input sequences as background control.

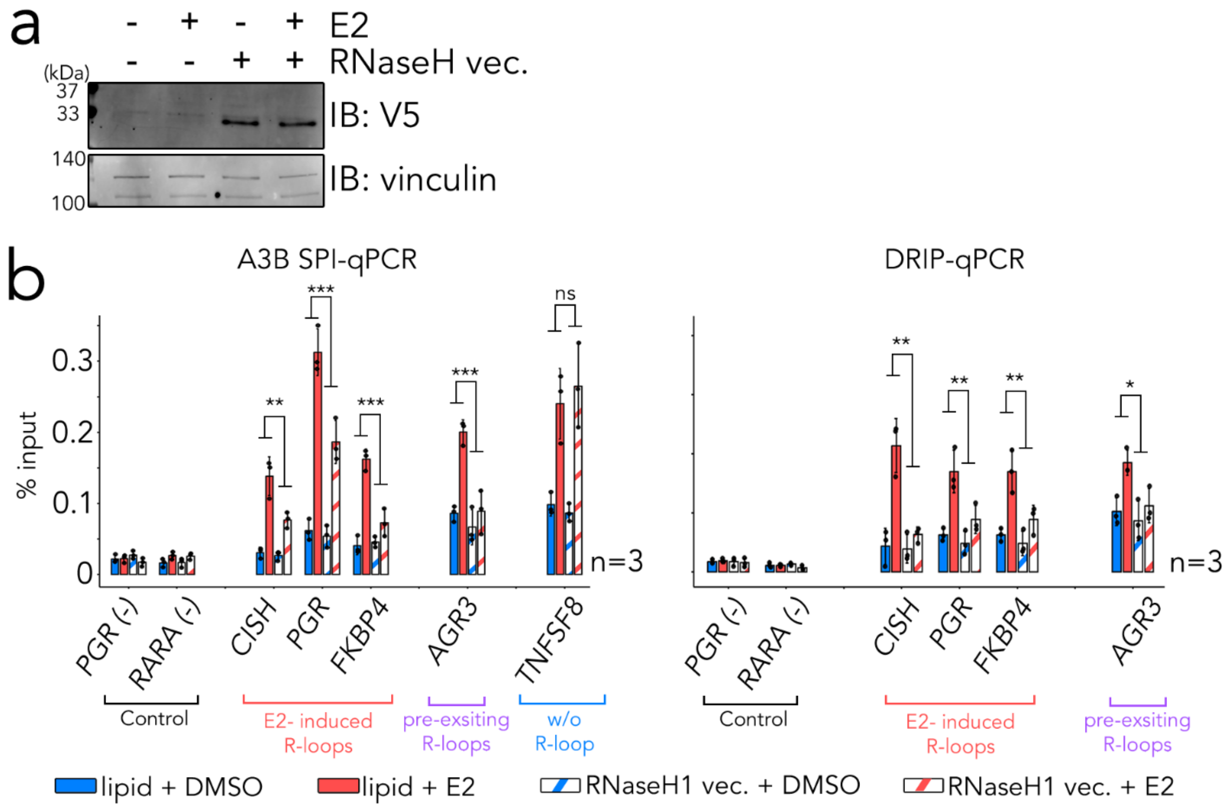

Supplementary Figure 17: R-loops induced by ER activation facilitates A3B binding in MCF7 cells.

a) Immunoblotting showing the overexpression of V5-tagged RNaseH1 in MCF7 cells. The immunoblot shown is representative of two independent experiments.

b) Bar graph showing Flag–A3B SPI–qPCR and DRIP–qPCR results in MCF7 cells. Data represent the mean of $n = 3$ biological replicates; error bars indicate SD. Two-way ANOVA was used to assess the effect of RNaseH1 treatment on the E2 response. For the left panel, $p = 6.5 \times 10^{-3}$, 1.6×10^{-3}, 9.0×10^{-4}, 9.0×10^{-4} and 7.825×10^{-1} for CISH, PGR, FKBP4, AGR3 and TNFSF8, respectively. For the right panel, $p = 2.4 \times 10^{-3}$, 9.5×10^{-3}, 9.8×10^{-3} and 2.32×10^{-2} for CISH, PGR, FKBP4 and AGR3, respectively. Asterisks indicate significance as follows: * $p < 0.05$, ** $p < 0.01$, *** $p < 1 \times 10^{-3}$, **** $p < 1 \times 10^{-4}$; ns, $p > 0.05$.

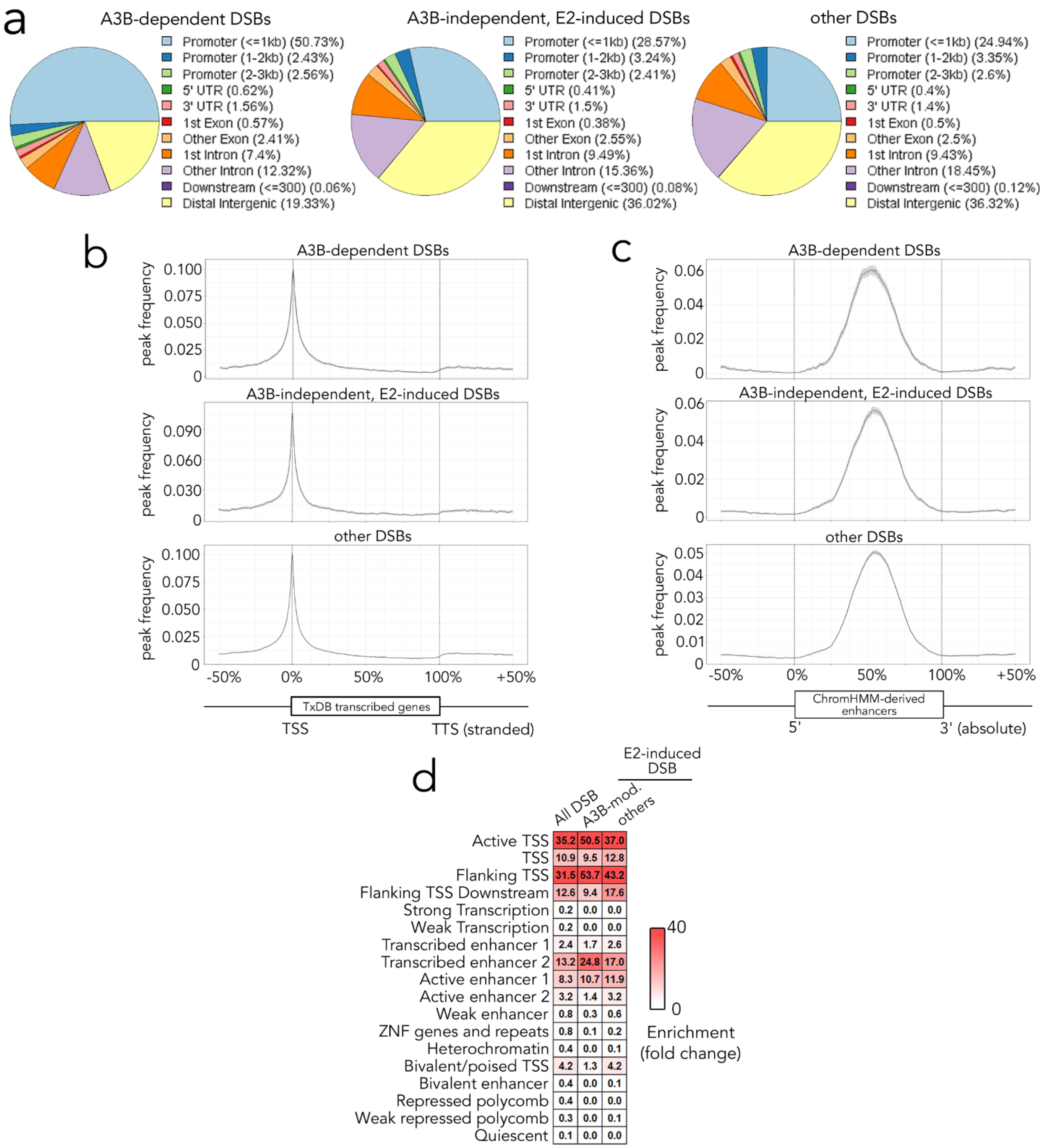

Supplementary Figure 18: Genome-wide analysis of the results from quantitative DSBCapture-seq.

a) Genomic annotation of peaks from quantitative DSBCapture-seq experiments.

b) Frequency of DSBCapure-seq peaks over transcribed gene body. Line denotes average value and shaded area for 95% CI.

c) Frequency of DSBCapure-seq peaks over ChromHMM-derived enhancers. Line denotes average value and shaded area for 95% CI.

d) Heat map showing enrichment scores for ChromHMM chromatin states at indicated DSBs.

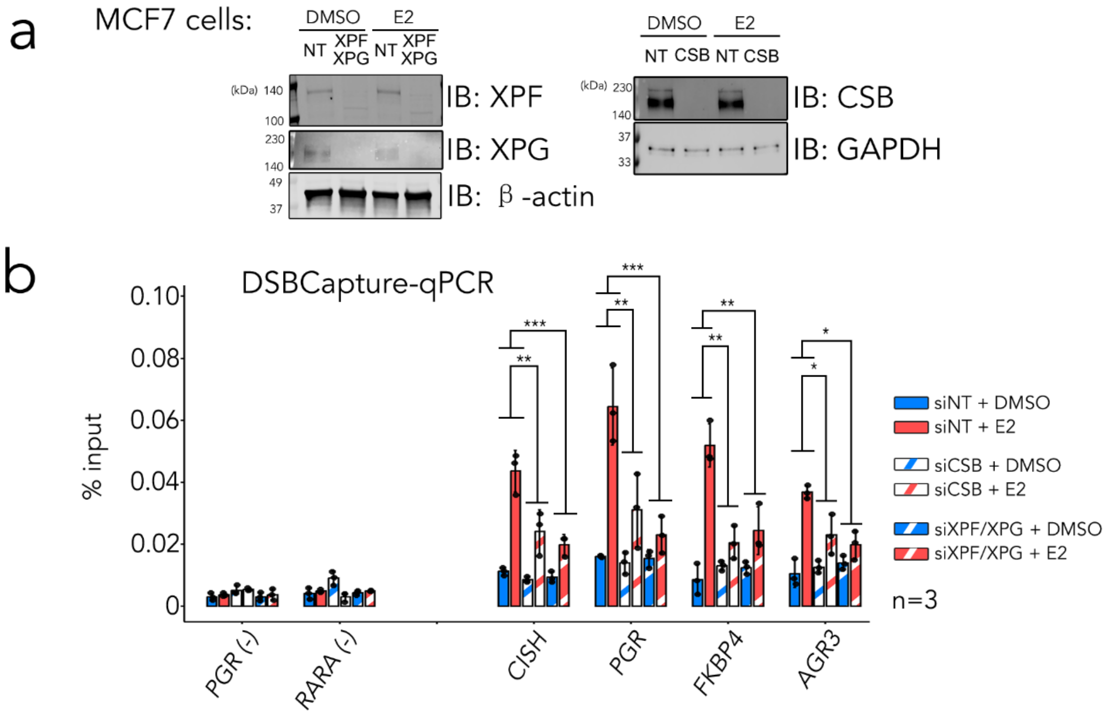

Supplementary Figure 19: Knockdown of TC-NER components reduces E2-induced A3B-dependent double-strand breaks in MCF7 cells.

a) Immunoblots of MCF7 cell lysates showing protein depletion following siRNA-mediated knockdown of XPF/XPG and CSB. Cells were transfected with non-targeting (NT) control or the indicated siRNAs for 48 h, followed by 2-hour stimulation with 100 nM E2 or DMSO vehicle. The immunoblot shown is representative of two independent experiments.

b) Bar graph quantification of DSBCapture qPCR signals at selected genomic loci. Data represent mean values from n = 3 biological repeats, and error bars indicate SD. Two-way ANOVA was used to assess the effect size of siRNA treatment on E2 response. For the effect of CSB depletion, ANOVA $p = 4.8 \times 10^{-3}$ (CISH), 8.4×10^{-3} (PGR), 1.8×10^{-3} (FKBP4), and 0.04 (AGR3). For the effect of XPF/XPG dual depletion, ANOVA $p = 5 \times 10^{-4}$ (CISH), 9×10^{-4} (PGR), 7.8×10^{-3} (FKBP4), and 0.01 (AGR3). *, **, and *** indicate $p < 0.05$, < 0.01, and $< 10^{-3}$, respectively.

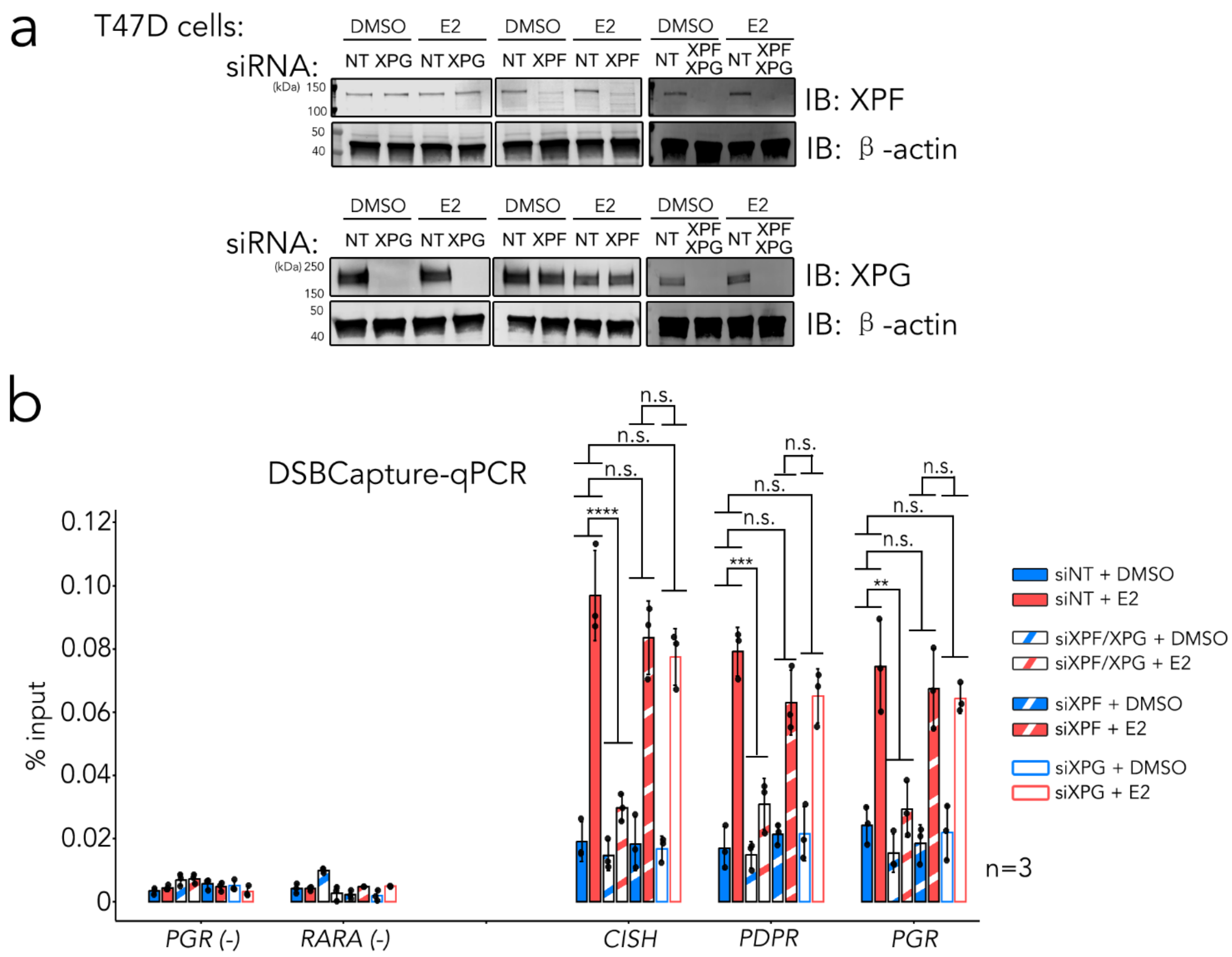

Supplementary Figure 20: TC-NER components contribute to conversion of A3B-modulated, E2-induced lesions into DNA double-strand breaks.

a) Immunoblots of T47D cell lysates showing protein depletion following siRNA-mediated knockdown of XPF and/or XPG. Cells were transfected with non-targeting (NT) control or the indicated siRNAs for 48 h, followed by 2-hour stimulation with 100 nM E2 or DMSO vehicle. The immunoblot shown is representative of two independent experiments.

b) Bar graph quantification of DSBCapture qPCR signals at selected genomic loci. Data represent mean values from n = 3 biological repeats, and error bars indicate SD. Two-way, two-tailed ANOVA was used to assess the effect size of siRNA treatment on E2 response. For the effect of dual XPG/XPF depletion, ANOVA $p = 8 \times 10^{-5}$ (CISH), 2×10^{-4} (PDPR), and 1.2×10^{-3} (PGR). *, **, ***, ****, and n.s. indicate $p < 0.05$, < 0.01, $< 10^{-3}$, $< 10^{-4}$, and > 0.05, respectively.

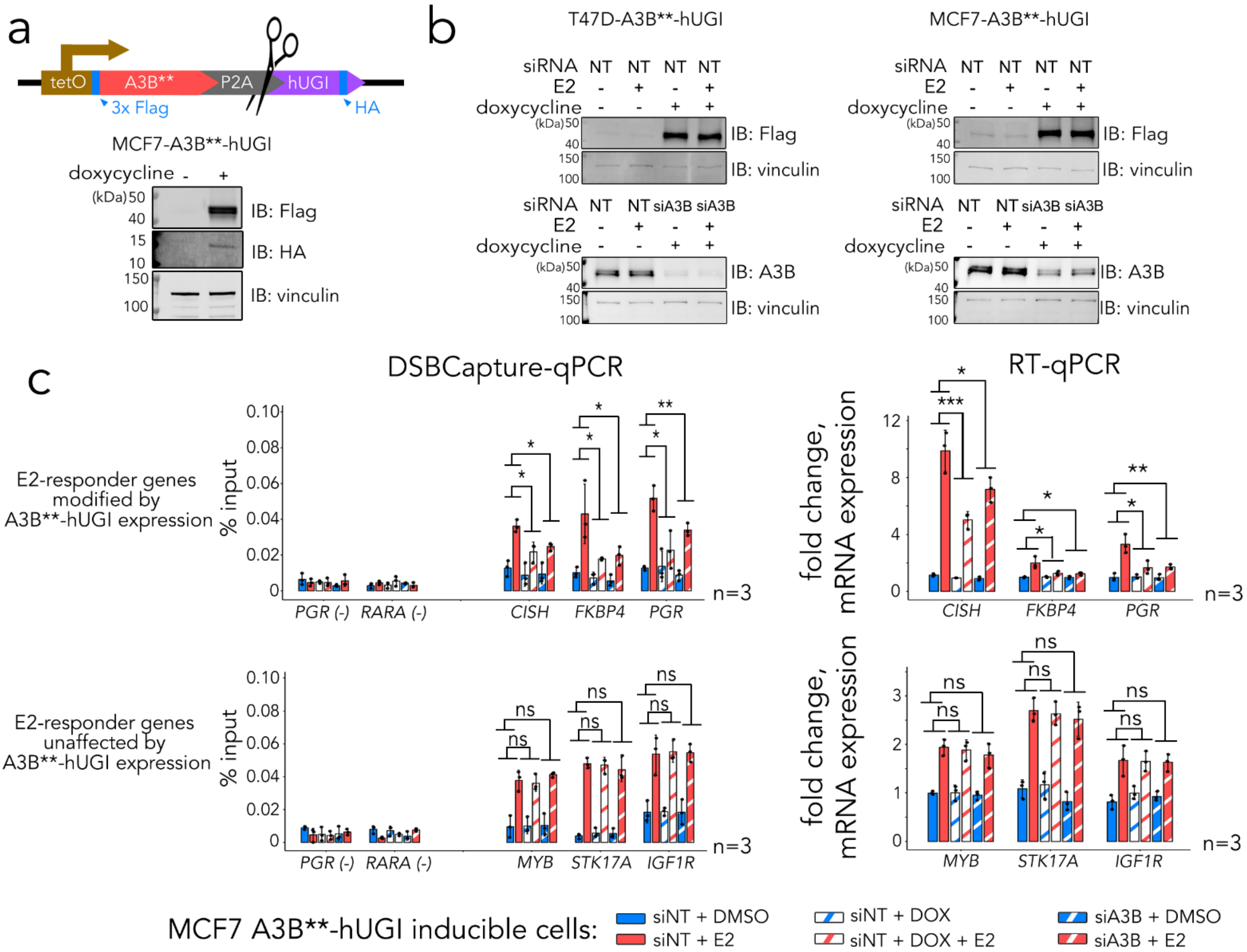

Supplementary Figure 21: Formation of A3B-modified DSBs promotes E2-regulated gene transcription.

a) Immunoblot analysis of MCF7 cells transduced with inducible A3B**-hUGI constructs, confirming expression of the indicated proteins. Cells were treated with1 µg/mL doxycycline for 24 h. The immunoblot shown is representative of two independent experiments.

b) Immunoblots of T47D and MCF7 cell lysates showing expression of lentiviral A3B**-hUGI and depletion of intrinsic A3B, cells were treated with NT/A3B siRNA or doxycycline for 48 hours before 2-hour stimulation by DMSO or 100 nM E2. The immunoblot shown is representative of two independent experiments.

c) Bar graph showing DSBCapture-qPCR results for the quantification of DSB formation (left), and RT-qPCR results for the quantification of the transcript level of associated genes (right). The experiment was conducted using MCF7 cells with a lentiviral inducible A3B**-hUGI cassette as shown in (b). Data represent mean values from $n = 3$ biological replicates, and error bars indicate SD. Two-way, two-tailed ANOVA was used to assess the effect size of doxycycline treatment or A3B siRNA on E2 response. For DSBCapture-qPCR, the effect of doxycycline induction yielded $p = 0.013$ (CISH), 0.022 (FKBP4), and 0.013 (PGR); the effect of A3B depletion yielded $p = 0.014$ (CISH), 0.027 (FKBP4), and 2.4×10^{-3} (PGR). For transcript levels, the effect of doxycycline induction yielded $p = 5 \times 10^{-4}$ (CISH), 0.024 (FKBP4), and 0.011 (PGR); the effect of A3B depletion yielded $p = 0.016$ (CISH), 0.018 (FKBP4), and 6.2×10^{-3} (PGR). *, **, ***, and n.s. indicate $p < 0.05$, < 0.01, $< 10^{-3}$, and > 0.05, respectively.

Supplemental Table

Supplemental Table 1. List of mutations on lentiviral A3B inducible expression vector for separated colonies.

Colony	Position on vector	Ref allele	Alt allele	Calculated fraction of Alt allele	Vector Feature	Impact on CDR	Outcome of mutation
M(+)-3	3383	C	T	0.027	Flag-APOBEC3B-P2A-hUGI,	p.(Leu104=)	Synonymous
M(+)-3	3679	C	T	0.023	Flag-APOBEC3B-P2A-hUGI,	p.(Phe202=)	Synonymous
A(-)-2	3836	G	C	0.017	Flag-APOBEC3B-P2A-hUGI,	p.(Ala254=)	Synonymous
M(+)-2	3836	G	C	0.037	Flag-APOBEC3B-P2A-hUGI,	p.(Ala254=)	Synonymous
M(+)-3	3836	G	C	0.036	Flag-APOBEC3B-P2A-hUGI,	p.(Ala254=)	Synonymous
M(+)-3	6258	T	A	0.007	rtTA3	p.(Gly19=)	Synonymous
M(+)-3	6401	T	C	0.028	rtTA3	p.(Phe67Tyr)	Non-synonymous
M(+)-3	6713	G	A	0.026	rtTA3	p.(Arg171Lys)	Non-synonymous
A(-)-2	6771	G	A	0.285	rtTA3	p.(Leu190=)	Synonymous
M(+)-2	6771	G	A	0.017	rtTA3	p.(Leu190=)	Synonymous
M(+)-1	7153	G	A	0.196	IRES	n.a.	n.a.
M(+)-3	7408	G	A	0.043	IRES	n.a.	n.a.
M(+)-3	7506	G	T	0.029	IRES	n.a.	n.a.
A(+)-4	7552	G	A	0.12	IRES	n.a.	n.a.
A(+)-5	7552	G	A	0.155	IRES	n.a.	n.a.
M(+)-3	7820	T	C	0.039	puromycin-N-acetyl-transferase	p.(Ser94Pro)	Non-synonymous
M(+)-1	7991	G	A	0.134	puromycin-N-acetyl-transferase	p.(Glu150=)	Synonymous
M(+)-3	8005	T	C	0.037	puromycin-N-acetyl-transferase	p.(Ala155=)	Synonymous
M(+)-1	8658	C	T	0.091	WPRE	n.a.	n.a.
A(+)-3	8716	G	T	0.173	WPRE	n.a.	n.a.
A(+)-4	8716	G	T	0.122	WPRE	n.a.	n.a.
A(+)-5	8716	G	T	0.159	WPRE	n.a.	n.a.
M(+)-1	8793	C	T	0.036	WPRE	n.a.	n.a.
M(+)-1	8850	G	A	0.076	None	n.a.	n.a.
A(+)-3	8857	G	T	0.176	None	n.a.	n.a.
A(+)-4	8857	G	T	0.129	None	n.a.	n.a.
A(+)-5	8857	G	T	0.133	None	n.a.	n.a.

Supplemental references

1 Zhang, Y. *et al.* Discovery of APOBEC cytidine deaminases inhibitors using a BspH1 restriction enzyme-based biosensor. *ChemistrySelect* **7**, e202201456, doi: https://doi.org/10.1002/slct.202201456 (2022).

2 Gao, T. *et al.* EnhancerAtlas: a resource for enhancer annotation and analysis in 105 human cell/tissue types. *Bioinformatics* **32**, 3543-3551, doi:10.1093/bioinformatics/btw495 (2016).

3 Chan, H. L. *et al.* Polycomb complexes associate with enhancers and promote oncogenic transcriptional programs in cancer through multiple mechanisms. *Nat Commun* **9**, 3377, doi:10.1038/s41467-018-05728-x (2018).
