# Supplementary material for: R-loop editing by DNA cytosine deaminase APOBEC3B modulates the activity of oestrogen receptor enhancers
Source: Nat Commun. 2026 Feb 18;17:2887. doi: 10.1038/s41467-026-69679-4 (PMC13031881; doi:10.1038/s41467-026-69679-4)
Supplement: Supplementary file 2 — Description of Additional Supplementary Files [file 41467_2026_69679_MOESM2_ESM.pdf]

**Description of Additional Supplementary Files**

**File Name:** Supplementary Data 1

**Description:** Genes with estradiol-responsive expression altered by hUGI in T-47D cells (RNA-seq).
