# Supplementary material for: R-loop editing by DNA cytosine deaminase APOBEC3B modulates the activity of oestrogen receptor enhancers
Source: Nat Commun. 2026 Feb 18;17:2887. doi: 10.1038/s41467-026-69679-4 (PMC13031881; doi:10.1038/s41467-026-69679-4)
Supplement: Supplementary file 4 — Reporting Summary [file 41467_2026_69679_MOESM4_ESM.pdf]

nature portfolio

Corresponding author(s): Paul A Clarke

Last updated by author(s): Jan 20, 2026

# Reporting Summary

Nature Portfolio wishes to improve the reproducibility of the work that we publish. This form provides structure for consistency and transparency in reporting. For further information on Nature Portfolio policies, see our Editorial Policies and the Editorial Policy Checklist.

## Statistics

For all statistical analyses, confirm that the following items are present in the figure legend, table legend, main text, or Methods section.

| n/a | Confirmed | |
|---|---|---|
| ☐ | ☒ | The exact sample size (*n*) for each experimental group/condition, given as a discrete number and unit of measurement |
| ☐ | ☒ | A statement on whether measurements were taken from distinct samples or whether the same sample was measured repeatedly |
| ☐ | ☒ | The statistical test(s) used AND whether they are one- or two-sided<br>*Only common tests should be described solely by name; describe more complex techniques in the Methods section.* |
| ☐ | ☒ | A description of all covariates tested |
| ☐ | ☒ | A description of any assumptions or corrections, such as tests of normality and adjustment for multiple comparisons |
| ☐ | ☒ | A full description of the statistical parameters including central tendency (e.g. means) or other basic estimates (e.g. regression coefficient) AND variation (e.g. standard deviation) or associated estimates of uncertainty (e.g. confidence intervals) |
| ☐ | ☒ | For null hypothesis testing, the test statistic (e.g. *F*, *t*, *r*) with confidence intervals, effect sizes, degrees of freedom and *P* value noted<br>*Give P values as exact values whenever suitable.* |
| ☒ | ☐ | For Bayesian analysis, information on the choice of priors and Markov chain Monte Carlo settings |
| ☒ | ☐ | For hierarchical and complex designs, identification of the appropriate level for tests and full reporting of outcomes |
| ☒ | ☐ | Estimates of effect sizes (e.g. Cohen's *d*, Pearson's *r*), indicating how they were calculated |

*Our web collection on statistics for biologists contains articles on many of the points above.*

## Software and code

Policy information about availability of computer code

| | |
|---|---|
| Data collection | Genome Aggregation Database (gnomAD) (Karczewski et al., 2020), UCSC Genome Browser (Kent et al., 2002), R-loop DB (Jenjaroenpun et al., 2017), Gene Expression Omnibus (GEO), ENCODE database. |
| Data analysis | Burrows-Wheeler Aligner (BWA) version 0.7.17 (Li and Durbin, 2009), Genome Analysis Toolkit (GATK, version 4.0.5) (DePristo et al., 2011), Picard Tools version 2.25.1, SNPiR software (Piskol et al., 2013), pBLAT algorithm (Wang and Kong, 2019), BEDTools version 2.30.0 (Quinlan and Hall, 2010), SAMtools version 1.12 (Li et al., 2009), Variant Effect Predictor (VEP) version 103 (McLaren et al., 2016), ChromHMM version 1.23 (Ernst and Kellis, 2017), MACS2 version 2.2.7.1 (Feng et al., 2012), MutationalPatterns version 3.2.0 (Blokzijl et al., 2018), Non-negative Matrix Factorisation (NMF) version 0.23.0 (Gaujoux and Seoighe, 2010), DESeq2 version 1.30.1 (Love et al., 2014), edgeR version 3.32.1 (Robinson et al., 2010), DiffBind version 3.0.15 (Ross-Innes et al., 2012), SeqPlots version 1.30.0 (Stempor and Ahringer, 2016), ComplexHeatmap version 2.6.2 (Gu et al., 2016), STAR aligner version 2.7.8a (Dobin et al., 2013), Rsubread version 2.4.3 (Liao et al., 2019), fGSEA version 1.16.0 (Subramanian et al., 2005), Integrative Genomics Viewer (IGV) version 2.9.4 (Robinson et al., 2011), GREAT analysis version 4.0.4 (McLean et al., 2010), Hyperclust (Mas-Ponte and Supek, 2020), G4Hunter (Bedrat et al., 2016), R language version 4.1.0 (R Core Team), ggplot2 version 3.3.5, qqman version 0.1.9 (Turner, 2018), MSPC version 6.0.0 (Jalili et al., 2015), Subread version 2.0.3, AME from MEME suite version 5.4.1 (Bailey et al., 2015; McLeay and Bailey, 2010), ChIPseeker version 1.26.2, COSMIC single-base substitution (SBS) signatures analysis |

For manuscripts utilizing custom algorithms or software that are central to the research but not yet described in published literature, software must be made available to editors and reviewers. We strongly encourage code deposition in a community repository (e.g. GitHub). See the Nature Portfolio guidelines for submitting code & software for further information.

## Data

Policy information about availability of data

All manuscripts must include a data availability statement. This statement should provide the following information, where applicable:

- Accession codes, unique identifiers, or web links for publicly available datasets
- A description of any restrictions on data availability
- For clinical datasets or third party data, please ensure that the statement adheres to our policy

The accession number for the WGS, ChIP-seq, SPI-seq, ssDRIP-seq, DSBCapture-seq and RNA-seq reported in this study at Gene Expression Omnibus (GEO) database is GSE193234. Processed results for RNA-seq are available in Supplemental Table 1. Previously published datasets used in this study include: GC-skew data for GRCh19 from R-loop DB [http://r-loop.org/]; T47D ER ChIP-seq data GSE148277 [https://www.ncbi.nlm.nih.gov/geo/query/acc.cgi?acc=GSE148277]; T47D GRO-seq data GSE128452 [https://www.ncbi.nlm.nih.gov/geo/query/acc.cgi?acc=GSE128452]; T47D RNAPII ChIP-seq data GSE105793 [https://www.ncbi.nlm.nih.gov/geo/query/acc.cgi?acc=GSE105793]; T47D Repli-Seq data ENCFF440QFG [https://www.encodeproject.org/files/ENCFF440QFG/]; T47D super enhancers data GSM2862201 [https://www.ncbi.nlm.nih.gov/geo/query/acc.cgi?acc=GSM2862201]; T47D Mnase-seq data GSE74308 [https://www.ncbi.nlm.nih.gov/geo/query/acc.cgi?acc=GSE74308].

## Research involving human participants, their data, or biological material

Policy information about studies with human participants or human data. See also policy information about sex, gender (identity/presentation), and sexual orientation and race, ethnicity and racism.

| | |
|---|---|
| Reporting on sex and gender | n/a |
| Reporting on race, ethnicity, or other socially relevant groupings | n/a |
| Population characteristics | n/a |
| Recruitment | n/a |
| Ethics oversight | n/a |

Note that full information on the approval of the study protocol must also be provided in the manuscript.

# Field-specific reporting

Please select the one below that is the best fit for your research. If you are not sure, read the appropriate sections before making your selection.

☒ Life sciences ☐ Behavioural & social sciences ☐ Ecological, evolutionary & environmental sciences

For a reference copy of the document with all sections, see nature.com/documents/nr-reporting-summary-flat.pdf

# Life sciences study design

All studies must disclose on these points even when the disclosure is negative.

| | |
|---|---|
| Sample size | The sample sizes for the experiments conducted in your study are summarized as follows: Whole-genome sequencing (WGS) involved five individual doxycycline-induced T47D clones (A+ colonies), three uninduced colonies (A– colonies), and three colonies expressing inactive A3B mutant (M+ colonies). For quantitative SPI-seq, two biological replicates per experimental condition were utilized. Quantitative ssDRIP-seq was performed using three biological replicates per condition. The DSBCapture-seq experiments included two biological replicates for each treatment group. RNA-seq experiments were conducted with four biological replicates per condition. Immunoprecipitation and quantitative PCR (qPCR) experiments generally comprised three biological replicates. Slot blot, immunoblotting, and co-immunoprecipitation experiments were consistently performed with at least two biological replicates. Statistical analyses were performed accordingly to accommodate these sample sizes.<br>Sample sizes were chosen based on established practice in the field and our previous work using similar assays, with the aim of achieving robust and reproducible effects while remaining within practical constraints of multi omic experiments. For cellular and biochemical experiments, we used at least three independent biological replicates with similar results, as stated in the figure legends. For cell based genomic assays (WGS, ChIP seq/SPI seq, ssDRIP seq, DSBCapture seq, RNA seq), we used 2–5 independent biological replicates per condition as specified in the figure legends and Methods, which is standard for these high dimensional datasets and sufficient to detect the large effect sizes observed. In all cases, replicate consistency and effect magnitude were used to justify that the chosen sample sizes were adequate. |
| Data exclusions | no data was excluded in this study. |
| Replication | All experiments involved in this study used biological replicates. |
| Randomization | All experiments involved in this study used fully random design. |

| | |
|---|---|
| Blinding | Allocation of samples into groups are blinded in all experiments. |

# Reporting for specific materials, systems and methods

We require information from authors about some types of materials, experimental systems and methods used in many studies. Here, indicate whether each material, system or method listed is relevant to your study. If you are not sure if a list item applies to your research, read the appropriate section before selecting a response.

## Materials & experimental systems

| n/a | Involved in the study |
|---|---|
| ☐ | ☒ Antibodies |
| ☐ | ☒ Eukaryotic cell lines |
| ☒ | ☐ Palaeontology and archaeology |
| ☒ | ☐ Animals and other organisms |
| ☒ | ☐ Clinical data |
| ☒ | ☐ Dual use research of concern |
| ☒ | ☐ Plants |

## Methods

| n/a | Involved in the study |
|---|---|
| ☐ | ☒ ChIP-seq |
| ☒ | ☐ Flow cytometry |
| ☒ | ☐ MRI-based neuroimaging |

## Antibodies

| | |
|---|---|
| Antibodies used | In this study, the following primary antibodies were used: anti-Flag tag (mouse monoclonal, clone M2, Sigma-Aldrich, cat. F1804; WB/IB 1:1,000; co-IP 1–2 μg per IP; ChIP/SPI 2–5 μg per IP), anti-HA tag (rabbit polyclonal, Proteintech, cat. 51064-2-AP; WB/IB 1:1,000), anti-GAPDH (rabbit polyclonal, Abcam, cat. ab9485; WB/IB 1:5,000), anti-APOBEC3B (rabbit monoclonal, clone EPR18138, Abcam, cat. ab184990; WB/IB 1:1,000), anti-APOBEC3B (rabbit polyclonal, Thermo Fisher Scientific, cat. PA5-11430; ChIP-qPCR 2–5 μg per IP), anti-V5 tag (mouse monoclonal, clone SV5-Pk1, Abcam, cat. ab27671; WB/IB 1:1,000), anti-Top1 (mouse monoclonal, Invitrogen, cat. MA5-32228; WB/IB 1:1,000), anti-α-tubulin (rabbit polyclonal, Abcam, cat. ab4074; WB/IB 1:5,000), anti-vinculin (mouse monoclonal, Abclonal, cat. A2752; WB/IB 1:2,000), anti-β-actin (mouse monoclonal, Abclonal, cat. AC026; WB/IB 1:5,000), anti-DNA:RNA hybrid S9.6 (mouse monoclonal, clone S9.6, Kerafast, cat. ENH001; ssDRIP 2–5 μg per IP, slot blot 1:1,000, co-IP 2–5 μg per IP), anti-CSB (goat polyclonal, clone E-18, Santa Cruz Biotechnology, cat. sc-10459; WB/IB 1:500–1:1,000), anti-XPF (rabbit polyclonal, Abcam, cat. ab76948; WB/IB 1:1,000), and anti-XPG (mouse monoclonal, clone 8H7, Santa Cruz Biotechnology, cat. sc-13563; WB/IB 1:500–1:1,000). Streptavidin-HRP (Pierce, Thermo Fisher Scientific, cat. 21130; WB and slot blot 1:5,000) was used for detection of biotinylated UdgX. HRP-conjugated secondary antibodies (anti-mouse IgG--HRP and anti-rabbit IgG--HRP, Cell Signaling Technology; typically 1:5,000 for WB/IB) were from Cell Signaling Technology. |
| Validation | All primary antibodies used in this study are commercially available and were validated by the manufacturers for the indicated applications, as documented on their product pages: anti-Flag tag (M2), Sigma-Aldrich/Merck, F1804: https://www.sigmaaldrich.com/US/en/product/sigma/f1804; anti-HA tag (polyclonal), Proteintech, 51064-2-AP: https://www.ptglab.com/products/HA-Tag-Antibody-51064-2-AP.htm; anti-GAPDH (polyclonal), Abcam, ab9485: https://www.abcam.com/gapdh-antibody-loading-control-ab9485.html; anti-APOBEC3B (EPR18138), Abcam, ab184990: https://www.abcam.com/apobec3b-antibody-epr18138-ab184990.html; anti-APOBEC3B (polyclonal), Thermo Fisher Scientific, PA5-11430: https://www.thermofisher.com/antibody/product/APOBEC3B-Antibody-Polyclonal/PA5-11430; anti-V5 tag (SV5-Pk1), Abcam, ab27671: https://www.abcam.com/v5-tag-antibody-sv5-pk1-ab27671.html; anti-Top1 (monoclonal), Invitrogen, MA5-32228: https://www.thermofisher.com/antibody/product/Topoisomerase-I-Antibody-Monoclonal/MA5-32228; anti-α-tubulin (polyclonal), Abcam, ab4074: https://www.abcam.com/alpha-tubulin-antibody-ab4074.html; anti-vinculin (monoclonal), Abclonal, A2752: https://www.abclonal.com/catalog-antibodies/Vinculin-Antibody-A2752; anti-β-actin (monoclonal), Abclonal, AC026: https://www.abclonal.com/catalog-antibodies/Anti-beta-Actin-Monoclonal-Antibody-AC026; anti-DNA:RNA hybrid (S9.6), Kerafast, ENH001: https://www.kerafast.com/item/enh001/anti-dnarna-hybrid-antibody-s96; anti-CSB (E-18), Santa Cruz Biotechnology, sc-10459: https://www.scbt.com/p/csb-antibody-e-18; anti-XPF (polyclonal), Abcam, ab76948: https://www.abcam.com/ercc4-xpf-antibody-ab76948.html; anti-XPG (8H7), Santa Cruz Biotechnology, sc-13563: https://www.scbt.com/p/xpg-antibody-8h7; Streptavidin-HRP, Pierce/Thermo Fisher Scientific, 21130: https://www.thermofisher.com/order/catalog/product/21130. All these antibodies were validated by the manufacturers for the listed applications (e.g., WB/IB, IP, ChIP, ssDRIP, slot blot), and we additionally confirmed expected band sizes and specific signal changes upon genetic manipulation in our own experiments, as described in the Methods and shown in the corresponding figures |

## Eukaryotic cell lines

Policy information about cell lines and Sex and Gender in Research

| | |
|---|---|
| Cell line source(s) | All cell lines used in this study are commercially available and were obtained from recognised repositories or vendors; all human breast cell lines are of female origin as specified by the providers. T47D human breast cancer cells (female; ductal carcinoma) were obtained from the American Type Culture Collection (ATCC, Manassas, VA, USA; ATCC catalog HTB-133; detailed information at https://www.atcc.org/products/htb-133). SK-BR-3 human breast cancer cells (female; adenocarcinoma) were obtained from ATCC (catalog HTB-30; https://www.atcc.org/products/htb-30). Lenti-X 293T packaging cells (human embryonic kidney–derived) were obtained from Clontech/Takara Bio (Lenti-X 293T Cell Line, catalog 632180; product page https://www.takarabio.com/products/gene-function/viral-transduction/lentivirus/packaging-systems-and-cells/lenti-x-293t-cells). |

| | |
|---|---|
| Authentication | None of the human cell lines used in this study (T47D, SK-BR-3 and Lenti-X 293T) are listed as cross-contaminated or misidentified in the International Cell Line Authentication Committee (ICLAC) database (https://iclac.org/databases/cross-contaminations/). All cell lines were authenticated by short tandem repeat (STR) profiling and the resulting STR fingerprints matched the reference profiles provided by the original vendors (ATCC for T47D, and SK-BR-3; Clontech/Takara for Lenti-X 293T). Cell stocks used for all assays were expanded from authenticated batches. |
| Mycoplasma contamination | All cell lines used in this study (T47D, SK-BR-3 and Lenti-X 293T) were routinely tested for mycoplasma contamination by quantitative PCR using mycoplasma-specific primer–probe sets, and all tests were negative throughout the course of the experiments. |
| Commonly misidentified lines (See ICLAC register) | n/a |

# Plants

| | |
|---|---|
| Seed stocks | n/a |
| Novel plant genotypes | n/a |
| Authentication | n/a |

# ChIP-seq

## Data deposition

☒ Confirm that both raw and final processed data have been deposited in a public database such as GEO.

☒ Confirm that you have deposited or provided access to graph files (e.g. BED files) for the called peaks.

| | |
|---|---|
| Data access links *May remain private before publication.* | https://www.ncbi.nlm.nih.gov/geo/query/acc.cgi?acc=GSE193234 |
| Files in database submission | https://www.ncbi.nlm.nih.gov/geo/query/acc.cgi?acc=GSE193234 |
| Genome browser session (e.g. UCSC) | n/a |

## Methodology

| | |
|---|---|
| Replicates | Whole-genome sequencing (WGS) was conducted using the BGISEQ-500 platform on five doxycycline-induced T47D colonies (A+ colonies), three uninduced control colonies (A– colonies), and three colonies expressing an inactive mutant A3B (M+ colonies). Strand-specific DNA:RNA immunoprecipitation sequencing (ssDRIP-seq) was performed with three biological replicates per experimental condition. Single-stranded DNA-associated protein immunoprecipitation sequencing (SPI-seq) experiments were performed in two biological replicates per condition. DSBCapture-seq, designed to detect DNA double-strand breaks (DSBs), was also conducted using two biological replicates per treatment group. RNA-seq analyses included four biological replicates per condition. Chromatin immunoprecipitation sequencing (ChIP-seq) experiments, including A3B binding and chromatin state analyses, generally included two biological replicates for quantitative assessments. Each sequencing approach provided detailed genomic insights, effectively addressing experimental aims relating to genomic mutations, transcriptional regulation, chromatin occupancy, DNA-RNA hybrid formation, and DNA damage. |
| Sequencing depth | For WGS, a minimum of 33X coverage was used; For ssDRIP-seq, 120 million reads for each of the samples; for DSBCapure-seq, SPI-seq and ChIP-seq minimum of 30 million for each of the samples. |
| Antibodies | Anti-Flag (Clone M2, F1804, Sigma-Aldrich), anti-S9.6 antibody for DNA:RNA hybrid detection (ENH001, Kerafast) |
| Peak calling parameters | For ssDRIP-seq:Peak calling was carried out using MACS2 package using pair-end fragment setting with input and ssDRIP samples. The resultant peaks were served as input for MSPC, and recurrent peaks were filtered using parameters '–w 1E-4 –s 1E-8 –c 4'.<br>Peaks on the same strand and within a distance of 1000 bp were merged using BEDTools, and converted to GTF format using UCSC tools (bedtogenepred and genepredtogtf).Read counting was conducted using Subread, and raw counts were analysed by DEseq2 using a generalized linear model with an interaction term.Genome_build: GRCh37<br>For SPI-seq:Peak calling was carried out using MACS2 package, where both input/IP sequence reads were used and the fragment length was set to 200 bp.Genome_build: GRCh37<br>For DSBCapture-seq:MACS2 was used to perform peak calling, with modelled narrow peak settings and an extended fragment length of 200 bp.For quantification, EdgeR analysis was performed using DiffBind.Genome_build: GRCh37 |

| | |
|---|---|
| Data quality | requirement of less than 5% FDR for peak calling and all quantification methods |
| Software | Sequencing data were processed and analysed using Burrows–Wheeler Aligner (BWA v0.7.17; Li and Durbin, 2009), BEDTools (v2.30.0; Quinlan and Hall, 2010), SAMtools (v1.15; Li et al., 2009), MACS2 (v2.2.7.1; Feng et al., 2012), DiffBind (v3.10.0; Ross-Innes et al., 2012), SeqPlots (v1.24.0; Stempor and Ahringer, 2016), Integrative Genomics Viewer (IGV v2.16.0; Robinson et al., 2011), MSPC (v4.0.2; Jalili et al., 2015), Subread (v2.0.3; Liao et al., 2013), AME from the MEME Suite (v5.5.5; Bailey et al., 2015; McLeay and Bailey, 2010) and ChIPseeker (v1.40.0; Yu et al., 2015). |
